# Little evidence for intralocus sexual conflict over the optimal intake of nutrients for life span and reproduction in the black field cricket *Teleogryllus commodus*

**DOI:** 10.1111/evo.13299

**Published:** 2017-07-12

**Authors:** James Rapkin, C. Ruth Archer, Charles E. Grant, Kim Jensen, Clarissa M. House, Alastair J. Wilson, John Hunt

**Affiliations:** ^1^ Centre for Ecology and Conservation, College of Life and Environmental Sciences University of Exeter Penryn Campus Penryn Cornwall TR10 9FE United Kingdom; ^2^ Department of Bioscience, Terrestrial Ecology Aarhus University Vejlsøvej 25 8600 Silkeborg Denmark; ^3^ School of Science and Health Western Sydney University Locked Bag 1797 Penrith NSW 2751 Australia; ^4^ Hawkesbury Institute for the Environment Western Sydney University Locked Bag 1797 Penrith NSW 2751 Australia

**Keywords:** Carbohydrate, Geometric Framework, intralocus sexual conflict, life span, nutrition, protein, reproduction

## Abstract

There is often large divergence in the effects of key nutrients on life span (LS) and reproduction in the sexes, yet nutrient intake is regulated in the same way in males and females given dietary choice. This suggests that the sexes are constrained from feeding to their sex‐specific nutritional optima for these traits. Here, we examine the potential for intralocus sexual conflict (IASC) over optimal protein and carbohydrate intake for LS and reproduction to constrain the evolution of sex‐specific nutrient regulation in the field cricket, *Teleogryllus commodus*. We show clear sex differences in the effects of protein and carbohydrate intake on LS and reproduction and strong positive genetic correlations between the sexes for the regulated intake of these nutrients. However, the between‐sex additive genetic covariance matrix had very little effect on the predicted evolutionary response of nutrient regulation in the sexes. Thus, IASC appears unlikely to act as an evolutionary constraint on sex‐specific nutrient regulation in *T. commodus*. This finding is supported by clear sexual dimorphism in the regulated intake of these nutrients under dietary choice. However, nutrient regulation did not coincide with the nutritional optima for LS or reproduction in either sex, suggesting that IASC is not completely resolved in *T. commodus*.

Sexual dimorphism in life span (LS) is widespread across the tree of life (Maklakov and Lummaa 2013; Austad and Fischer 2016). For example, in many species of insects (e.g., Promislow 2003; Bonduriansky et al. 2008; Austad and Fischer 2016) and mammals (e.g., Austad 1997; Promislow 2003; Clutton‐Brock and Isvaran 2007), including humans (Austad 2006; Maklakov et al. 2008), females typically live longer than males, whereas the reverse pattern appears commonplace across species of nematodes (McCulloch and Gems 2003) and birds (Promislow 1992; Donald 2007; Tower and Arbeitman 2009). Understanding the reasons for these sex differences in LS has been the topic of much debate and continues to puzzle biogerontologists and evolutionary biologists (Maklakov and Lummaa 2013; Austad and Fischer 2016). Early biological explanations viewed sexual dimorphism in LS and ageing as a maladaptation resulting from the asymmetric inheritance of sex chromosomes (the “unguarded X (or Z)” hypothesis, Tower 2006) or mitochondrial genomes (the “mother's curse” hypothesis, Frank and Hurst 1996; Gemmell et al. 2004; Camus et al. 2012) that lead to increased mortality in the shorter lived sex. Although these processes have received considerable empirical support, it is clear that their ability to explain the observed taxonomic patterns of sex differences in LS is incomplete at best (Maklakov and Lummaa 2013; Austad and Fischer 2016). More recently, the role of sexual selection in the evolution of sexual dimorphism in LS and rates of ageing has received considerable attention, raising the possibility that these sex differences may be an adaptive response to how the sexes optimize the trade‐off between LS and reproduction, shaped by sexual selection (Graves 2007; Bonduriansky et al. 2008; Maklakov and Lummaa 2013; Austad and Fischer 2016).

In most sexually reproducing species, one sex (typically males) produces numerous, small gametes that compete for access to larger, less abundant gametes produced by the other (typically females). This divergence in reproductive investment means that male fitness is generally more variable than female fitness and this increases both the opportunity for, and intensity of, sexual selection in males (Bateman 1948; Trivers 1972). Consequently, males are expected to pursue a high risk, “live fast, die young,” strategy that has the potential to yield high fitness returns over a short time scale (Graves 2007; Bonduriansky et al. 2008) and both theoretical models (e.g., Kokko, 1997, 2001) and empirical data (e.g., Hunt et al. 2004; Robinson et al. 2006) show that this can be achieved by trading LS for enhanced mating success. In contrast, female fitness is limited by the amount of time available to accrue the necessary resources for offspring production and so viability selection acting on females is expected to promote a low risk, “low wear and tear” strategy that yields moderate rates of return over a longer time period (Graves 2007; Bonduriansky et al. 2008). Sexual selection is, therefore, expected to promote the evolution of a shorter LS and more rapid ageing in males than females (Graves 2007; Bonduriansky et al. 2008). However, while comparative (e.g., Promislow 1992; Clutton‐Brock and Isvaran 2007) and experimental evolution (e.g., Maklakov et al. 2008) studies have provided empirical support for this general prediction, this pattern is far from universal. In particular, in species where reproductive effort increases with age in males but not in females and this enhances male mating success, sexual selection may actually favor the reverse pattern whereby males live longer and age more slowly than females (e.g., Archer et al. 2012).

This divergence in the reproductive strategies of the sexes is also expected to promote the sex‐specific allocation of key resources to reproduction and LS (Bonduriansky et al. 2008). Indeed, a link among reproductive effort, LS, and nutritional resources has been shown in numerous empirical studies by restricting dietary intake (Mair and Dillin 2008). Modest dietary restriction (a reduction in food intake without malnutrition), has been shown to extend LS across a wide range of animal taxa, spanning from yeast (e.g., Jiang et al. 2002) to primates (e.g., Colman et al. 2009), and meta‐analysis across 36 species has shown that this effect is typically more pronounced in females than males (Nakagawa et al. 2012). The effects of dietary restriction on LS have traditionally been attributed to caloric restriction (e.g., Partridge and Brand 2005; Masoro 2006), with the sex differences explained by the divergence in the energetic costs of reproduction (Barnes and Partridge 2003; Bonduriansky et al. 2008). In females, the extension of LS through caloric restriction is explained by the associated reduction in fecundity, which enables more resources to be allocated to somatic maintenance (Partridge and Brand 2005). In contrast, the energetic demands of reproduction are considered to be lower in males than females, weakening the trade‐off between LS and reproduction under caloric restriction (Bonduriansky et al. 2008). A common limitation of caloric restriction studies, however, is that they typically do not separate the effects of caloric intake from the intake of specific nutrients on LS and reproduction.

A multidimensional nutritional framework, known as the Geometric Framework for nutrition (Simpson and Raubenheimer 2012), provides a solution to this issue by restricting animals to an array of diets that differ in both nutrient composition and caloric content. Importantly, studies using the Geometric Framework have shown that it is the intake of specific nutrients and not calories per se that mediates the trade‐off between reproduction and LS and is ultimately responsible for extending LS (e.g., Lee et al. 2008; Maklakov et al. 2008; Fanson et al. 2009; Fanson and Taylor 2012; Harrison et al. 2014; Solon‐Biet et al. 2014; Jensen et al. 2015). For example, LS is maximized at a high intake of nutrients (and therefore calories) with a low protein (P) to carbohydrate (C) ratio (P:C) in a range of invertebrate species, including *Drosophila melanogaster* (Lee et al. 2008; Jensen et al. 2015), the Queensland fruit fly *Bactrocera tryoni* (Fanson et al. 2009; Fanson and Taylor 2012), and the field crickets *Teleogryllus commodus* (Maklakov et al. 2008) and *Gryllus veletis* (Harrison et al. 2014). This general pattern is also consistent with the results of a meta‐analysis showing that P restriction was more effective in extending LS than caloric restriction across a diverse range of animal species (Nakagawa et al. 2012).

Given the success of the Geometric Framework in partitioning the effects of calories and specific nutrients (Simpson and Raubenheimer 2012), it is surprising that the effect of nutrient intake on LS and reproduction has only been formally compared across the sexes in three species: *T. commodus* (Maklakov et al. 2008), *G. veletis* (Harrison et al. 2014), and *D. melanogaster* (Jensen et al. 2015). Despite this, a number of clear patterns have emerged from these studies on the differential effects that P and C intake has on these traits in the sexes. First, the sex differences in the effects of P and C intake on LS in the sexes are small. In *T. commodus* and *G. veletis*, female LS is maximized at 1_P_:8_C_, whereas male LS is maximized at 1_P_:5_C_ and 1_P_:3_C_, respectively (Maklakov et al. 2008; Harrison et al. 2014). In both species, however, the nutritional maxima for LS in the sexes overlap suggesting that this difference in P:C ratio is small. In *D. melanogaster*, LS was maximized at 1_P_:16_C_ in both sexes (Jensen et al. 2015). Second, divergence in the effects of P and C intake on reproduction in the sexes is much larger than observed for LS. In all three insect species, females require a much higher intake of P to maximize egg production (1_P_:1_C_ in *T. commodus*, 3_P_:1_C_ in *G. veletis*, 1_P_:2_C_ in *D. melanogaster*) relative to males that require a higher intake of C to maximize calling effort (1_P_:8_C_ in *T. commodus*, 1_P_:3_C_ in *G. veletis*) or the number of offspring sired during competition (1_P_:16_C_ in *D. melanogaster*) (Maklakov et al. 2008; Harrison et al. 2014; Jensen et al. 2015). Finally, as females in these species are unable to maximize LS and reproduction at the same intake of nutrients, there is a strong nutritionally based trade‐off between these two traits. In contrast, the smaller divergence in the nutrient intake maximizing LS and reproduction means that this nutritionally based trade‐off is less pronounced in males.

Optimal foraging theory predicts that animals will evolve foraging mechanisms that maximize their fitness (Stephens and Krebs 1986). Given the observed sexual divergence in the nutrients required to maximize LS and reproduction in the sexes, males and females should, therefore, be expected to regulate their intake of P and C in different ways when given dietary choice. This appears to be the case for *G. veletis*, where females regulate their intake of nutrients to a slightly higher P:C ratio than males (1_P_:2.31_C_ in females, 1_P_:4.1_C_ in males; Harrison et al. 2014). However, males and females regulate their nutrient intake to the same P:C ratio in both *T. commodus* (1_P_:2.96_C_; Maklakov et al. 2008) and *D. melanogaster* (1_P_:4_C_; Jensen et al. 2015). Furthermore, the regulated intake point (RIP) for males and females in all three species, which is defined as the point in nutrient space that individuals actively defend when given dietary choice (Raubenheimer and Simpson 1993), do not match the sex‐specific nutritional optima for either LS or reproduction (Maklakov et al. 2008; Harrison et al. 2014; Jensen et al. 2015). Collectively, this evidence suggests that the sexes are constrained from regulating their intake of nutrients to optimize these traits. One possible constraint that has received recent attention is intralocus sexual conflict (IASC) over the optimal intake of nutrients (Maklakov et al. 2008; Maklakov and Lummaa 2013; Reddiex et al. 2013; Jensen et al. 2015).

In general, IASC occurs because many sexually homologous (or shared) traits are subject to contrasting patterns of selection but have a common genetic basis in the sexes (Lande 1980; Bonduriansky and Chenoweth 2009). This generates an evolutionary “tug‐of‐war” between the sexes that can prevent one or both sexes from reaching their sex‐specific phenotypic optima and the evolution of sexual dimorphism in the shared trait(s) (Lande 1980; Bonduriansky and Chenoweth 2009). However, the lack of sexual dimorphism in nutrient regulation and the suboptimal intake of nutrients for LS and reproduction observed in *T. commodus* (Maklakov et al. 2008), *G. veletis* (Harrison et al. 2014), and *D. melanogaster* (Jensen et al. 2015) does not necessarily provide clear evidence that IASC over the optimal intake of nutrients is currently operating in the population (Bonduriansky and Chenoweth 2009). More definitive evidence requires showing that there are both sex differences in the effect of nutrient intake on LS and reproduction and strong and positive intersexual genetic correlations (*r*_MF_) for the intake of nutrients under dietary choice, which indicate that nutrient intake is not free to evolve independently in the sexes (Bonduriansky and Chenoweth 2009). Consequently, while these existing studies clearly satisfy the first criterion by showing the differential effects of P and C intake on LS and reproduction in the sexes, by not estimating *r*_MF_ for P and C intake when individuals are provided with dietary choice, they are unable to conclusively demonstrate that IASC over the optimal intake of nutrients for LS or reproduction is occurring. To date, only a single study on *D. melanogaster* has examined both of these criteria, but found little scope for IASC over the optimal intake of P and C for reproduction in this species (Reddiex et al. 2013). This conclusion was largely based on the fact that the observed sex differences in the effects of nutrient intake on reproduction were relatively minor and *r*_MF_ for P intake did not differ statistically from zero (Reddiex et al. 2013). However, it is possible that the relative minor divergence in nutritional effects across the sexes reflects the fact that nutrient intake and reproductive performance were only measured over a very short time period (four days) rather than across the lifetime of individuals. Moreover, by examining dietary choice over a restricted range (a single diet pair) rather than multiple diet pairs, it is possible that estimates of *r*_MF_ in this study are biased. Consequently, the verdict is still very much out on the importance of IASC over the optimal intake of nutrients in the evolution of sex differences in LS and reproduction.

In this study, we perform three experiments using the Geometric Framework to formally document the existence and directly quantify the strength of IASC over the optimal intake of P and C for LS and reproduction in male and female *T. commodus*. In Experiment 1, to characterize the sex‐specific effects of nutrients on LS and reproduction, we quantify the linear and nonlinear effects of P and C intake on LS, daily reproductive effort (DRE), and lifetime reproductive effort (LRE) in males and females by restricting individuals to one of 24 different holidic diets and construct nutritional landscapes to help visualize these effects. We then formally compare these nutritional effects to determine if the intakes of P and C have different effects on these traits in the sexes. In Experiment 2, we test whether the between‐sex genetic architecture for nutrient regulation constrains the evolution of sex‐specific nutritional optima, by examining the quantitative genetics of nutrient regulation within and between the sexes using a feeding choice experiment (incorporating four alternate diet pairs) within a half‐sib breeding design. We combine these genetic estimates with the effects of P and C on LS, DRE, and LRE in the sexes to directly quantify the strength of IASC over the optimal intake of these nutrients. Finally, in Experiment 3 we conduct a second choice feeding experiment using male and female crickets from our outbred population to determine whether the sexes regulate their intake of P and C differently under dietary choice. We calculate the RIP for each sex and then map these estimates onto the nutritional landscapes from Experiment 1 to determine if nutrient regulation under dietary choice is optimal for LS, DRE, and/or LRE in the sexes.

## Material and Methods

### ANIMALS AND HUSBANDRY

The *T. commodus* used in this study were collected from the wild in March 2009 from Smith's Lake, New South Wales, Australia and used to establish a panmictic laboratory population, which has been maintained in large cultures of approximately 500 animals for 10 nonoverlapping generations. Laboratory cultures are kept in 110 L boxes at 28°C ± 1°C, under a 13‐h light:11‐h dark cycle, cleaned weekly and provided with cardboard egg carton for shelter, water ab libitum in 50 mL test tubes plugged with cotton wool, egg pads consisting of damp cotton wool in a Petri dish and a 50% mixture of cat food (Purina Go Cat Senior^©^, St Louis, MO) and rat food (SDS Diets, Essex, UK; 24.82% P, 30.45% C).

### ARTIFICIAL DIETS AND FEEDING PROTOCOL

We made 24 artificial, powdered diets that varied in P:C ratio, as well as overall caloric content, based on the established protocol outlined by Simpson and Abisgold (1985) and used previously (South et al. 2011; Rapkin et al. 2016). The composition and distribution of these diets in nutritional space can be found in the electronic supplementary material (Appendix S1 and Fig. S1).

Experimental animals were given either one (Experiment 1) or two (Experiments 2 and 3) dishes of diet of measured dry weight. Water was provided ad libitum in a 5 mL test tube plugged with cotton wool. Food was provided in feeding platforms constructed by gluing the upturned lid of a vial (1.6 cm diameter, 1.6 cm deep) onto the middle of a Petri dish (5.5 cm diameter). Any diet spilled during feeding was collected in the Petri dish and weighed. All diets were dried in an oven (Binder FD115) at 30°C for 72 h before weighing. Feeding platforms with diet were weighed before and after each feeding period using an electronic balance. Feces were removed from the diet and feeding platform using forceps prior to re‐weighing. Diet consumption was calculated as the difference in dry weight of diet before and after feeding. This amount of consumed diet was converted to a weight of ingested P and C by multiplying by the proportion of these nutrients in the diet (see South et al. 2011).

### EXPERIMENT 1: THE EFFECT OF NUTRIENT INTAKE ON LIFE SPAN AND REPRODUCTIVE EFFORT IN THE SEXES

To determine the effects of P and C intake on male and female LS and reproductive effort, 10 males and 10 females were established at random on each of the 24 diets on their day of eclosion. However, some crickets escaped or died prematurely (due to accidental death) and were excluded from the final analysis (total: males *n* = 208; females *n* = 222). Food of known dry mass was provided every three days and on the evening of day 6 post eclosion, the feeding platform was removed and a stock animal of the opposite sex was introduced to the container. This mate was removed on day 7 when new food was provided and this weekly cycle was repeated throughout the experimental animal's lifetime. Animals were checked for mortality daily.

The reproductive effort of males and females was measured on the evening of day 7 and once a week thereafter. To quantify female reproductive effort, females were provided with a small Petri dish (5‐cm diameter) full of moist sand for oviposition for a seven‐day period, after which it was removed and frozen at −20°C for storage and replaced with a fresh dish of moist sand. To count eggs, the contents of each Petri dish were emptied into a container of water and the eggs removed with fine forceps and counted. Male reproductive effort was measured as the amount of time spent calling each night (hereafter referred to as calling effort), using a custom‐built electronic monitoring device as described in full detail in Archer et al. (2012). Calling effort has been shown to be a good measure of reproductive effort in *T. commodus* because calling is metabolically expensive to produce (Kavanagh 1987), and females have been shown to prefer males that call more (Bentsen et al. 2006).

#### Statistical analysis

We quantified the linear and nonlinear (quadratic and correlational) effects of P and C intake on LS, DRE, and LRE using a multivariate response surface approach (Appendix S2; but see South et al. 2011). Prior to analysis, P and C intake and all response variables (RVs) were transformed to a mean of zero and SD of one using a *Z*‐transformation. Nonparametric thin‐plate splines were used to visualize the nutritional landscape for each RV and were constructed using the *Tps* function in the “FIELDS” package (Nychka et al. 2015) of R (R Core Team, version 3.1.2, Vienna, Austria, www.r-project.org).

We used a sequential model‐building approach to determine whether the linear and nonlinear effects of P and C intake differed across our RVs (South et al. 2011). Full details of this approach are outlined in the electronic Supporting Information (Appendix S3). Although the sequential approach provides a statistical test of the difference in magnitude of the linear and nonlinear gradients across RVs, it does not provide information on the direction of this difference in nutrient space. As such, it is possible that RVs show differences in the magnitude of linear and nonlinear gradients, even though the optimal expression of these traits resides in a similar location in nutrient space. To account for this, we also calculated the angle (**θ**) between linear nutritional vectors for the two RVs being compared using the trigonometry procedure outlined in Bunning et al. (2015) as:

(1) θ= co s−1a·b∥a∥·∥b∥,

where arepresents the linear effects of P and C intake on the first RV being compared, brepresents the linear effects of these nutrients on the second RV, $∥a∥\;=\;\sqrt{a\cdot a}\;$and$\;∥b∥\;=\;\sqrt{b\cdot b}$. Whenθ= 0°, the vectors are perfectly aligned and the optima for the two RVs being compared reside in the same region in nutrient space, whereas θ= 180° represents the maximum possible divergence between vectors. We estimated the 95% credible interval (CI) for θ using a Bayesian approach implemented in the “MCMCglmm” package (Hadfield 2016) of R. For each RV, we ran a separate linear model ($\mathrm{RV}\;=\beta_{1}\;P+\beta_{2}C+\varepsilon$) using 400,000 Markov chain iterations with a burn‐in of 20,000, a thinning interval of 25 and a relatively uninformative prior (*V* = 1, ν = 0.02), to create a posterior distribution of β. We used these distributions in equation (1) to generate 15,200 values for θ. We used the posterior mean of these values as our parameter estimate of θand the highest posterior density (HPD) interval to estimate the 95% CIs of θ, both implemented in the *HPDinterval* function of the ”MCMCglmm” package. The associated R code for this procedure is provided in the electronic Supporting Information (Appendix S4).

### EXPERIMENT 2: THE QUANTITATIVE GENETICS OF NUTRIENT REGULATION

To estimate the quantitative genetics of nutrient regulation, we used a split‐brood half‐sib breeding design whereby sons and daughters from each full‐sib family were split across four different diet pairs and their intake of nutrients measured under dietary choice for 21 days. The half‐sib breeding design was established by mating each of 30 randomly chosen virgin sires with three randomly chosen virgin dams. A total of 50 offspring from each dam were collected and reared in a family group in an individual plastic container (10 cm × 10 cm × 5 cm) for three weeks, with access to an *ad libitum* supply of ground cat food (Purina Go Cat Senior^©^) and water provided in a 5‐cm plastic tube plugged with cotton wool. After three weeks, 12 sons and 12 daughters per dam were isolated and established in individual plastic containers (5 cm x 5 cm x 5 cm) and provided with ad libitum cat food pellets and water and checked daily for eclosion to adulthood. Containers were cleaned and fresh food and water were provided weekly.

On the day of eclosion, we randomly allocated three sons and three daughters, per dam, to each of four different diet pairs (total *n* = 2160). The diets chosen to form the four diet pairs were diets 2, 4, 22, and 24 (pair 1: diets 2 and 22; pair 2: diets 2 and 24; pair 3: diets 4 and 22; pair 4: diets 4 and 24), and are the same diets as those used in the choice feeding experiments of Bunning et al. (2016) and Rapkin et al. (2016). These diet pairs provide a broad coverage in nutrient space (Fig. S1). Dietary intake was measured every three days for a total of 21 days (i.e., seven feeding periods) with fresh diet being provided at each measurement period. All experimental crickets were mated to a virgin cricket of the opposite sex (taken at random from the outbred culture) on the evening of day 8 post eclosion and this mating partner was removed the following morning.

#### Statistical analysis

We estimated the additive genetic variance–covariance matrix (**G**) and corresponding estimates of narrow sense heritability (*h* ^2^) and additive genetic correlations for the regulated intake of P and C within (*r*_M_ and *r*_F_) and between the sexes (*r*_MF_) using a multivariate animal model (four variables: two sexes with two nutrients [P and C] per sex) (Wilson et al. 2010) implemented in the “MCMCglmm” package of R, with the mean of each variable fitted as a fixed effect in the model and the additive genetic effect for each variable fitted as a random effect. The model was run for 250,000 iterations with a burn‐in of 100 and a thinning interval of 50, and using a relatively uninformative prior (*V* = diag(4), ν = 1.002). It is important to note that the above linear equation does not contain diet pair and, therefore, the resulting genetic parameters are estimated across diet pairs. Consequently, our genetic parameters are for the RIP of P and C, which is typically calculated as the mean intake of these nutrients across diet pairs (Raubenheimer and Simpson 1993).

We estimated the predicted evolutionary response of the regulated intake of P and C in males and females using the following equation (Lande 1980):

(2) $$\Delta \;\bar{z}=\frac{1}{2}\;\beta G$$

where $\Delta \bar{z}$ represents the vector of predicted responses of the regulated intake of P and C in males ($\Delta {\bar{z}}_{m}$) and females ($\Delta {\bar{z}}_{f}$), respectively:

(3) $$\Delta \;\bar{z}=\left(\begin{array}{c} \Delta {\bar{z}}_{m}\\ \Delta {\bar{z}}_{f} \end{array}\right)\;$$

and β represents the vector of linear nutritional effects for males ($\beta_{m}$) and females ($\beta_{f}$), respectively (taken from Table 1):

(4) $$\beta \;=\left[\begin{array}{c} \beta_{f}\\ \beta_{m} \end{array}\right]\;$$

**Table 1:** Linear and nonlinear effects of protein (P) and carbohydrates (C) on life span (LS), daily reproductive effort (DRE), and lifetime reproductive effort (LRE) in female (A) and male (B) *Teleogryllus commodus*

	Linear effects		Nonlinear effects		
Response variables	P	C	P × P	C × C	P × C
(A) Females					
LS					
Coefficient ± SE	−0.06 ± 0.06	0.60 ± 0.06	−0.08 ± 0.05	−0.32 ± 0.05	−0.33 ± 0.08
*t*	1.10	10.46	1.53	5.99	4.13
df	219	219	216	216	216
*P*	0.27	0.0001	0.13	0.0001	0.0001
DRE					
Coefficient ± SE	0.52 ± 0.06	0.53 ± 0.06	−0.35 ± 0.05	−0.09 ± 0.05	0.21 ± 0.08
*T*	8.58	8.59	6.48	1.67	2.58
Df	219	219	216	216	216
*P*	0.0001	0.0001	0.0001	0.09	0.011
LRE					
Coefficient ± SE	0.35 ± 0.06	0.70 ± 0.06	−0.33 ± 0.05	−0.18 ± 0.05	0.06 ± 0.08
*T*	6.09	12.29	6.41	3.49	0.75
Df	219	219	216	216	216
*P*	0.0001	0.0001	0.0001	0.001	0.45
(B) Males					
LS					
Coefficient ± SE	0.28 ± 0.06	0.76 ± 0.06	−0.25 ± 0.03	−0.22 ± 0.04	−0.19 ± 0.06
*T*	4.89	13.35	7.44	5.37	3.03
Df	205	205	202	202	202
*P*	0.0001	0.0001	0.0001	0.0001	0.003
DRE					
Coefficient ± SE	−0.15 ± 0.04	0.80 ± 0.04	−0.01 ± 0.02	−0.11 ± 0.03	−0.25 ± 0.05
*T*	3.80	20.84	0.31	3.78	4.60
Df	205	205	202	202	202
*P*	0.0001	0.0001	0.76	0.0001	0.0001
LRE					
Coefficient ± SE	−0.09 ± 0.04	0.79 ± 0.04	−0.04 ± 0.03	−0.10 ± 0.04	−0.22 ± 0.05
*T*	2.17	18.42	1.33	2.93	4.32
Df	205	205	202	202	202
*P*	0.03	0.0001	0.18	0.004	0.001

DRE and LRE were measured as egg production and calling effort in females and males, respectively.

**G** represents the additive genetic variance–covariance matrix that can be partitioned into four submatrices, following Lande (1980):

(5) $$G\;=\left[\begin{array}{cc} G_{m} & \;B\\ B^{T} & \;G_{f} \end{array}\right]\;$$

where $G_{m}\;$and $G_{f\;}$are the within‐sex additive genetic variance–covariance matrix for males and females, respectively, while **B** (and its transpose, $B^{T}$) are the between‐sex additive genetic covariance matrices that ultimately determine the extent to which the sexes are able to evolve independently. The constant ½ appears because both parents are assumed to make equal autosomal contributions to the offspring of both sexes (Lande 1980). As $\beta_{m}\;$and $\beta_{f}$ measure the slope of the linear regression of P and C intake on a RV, and we examined the effects of nutrients on three RVs (LS, DRE, and LRE), there are three estimates of β and therefore $\Delta \bar{z}\;$for each sex. We estimated the 95% CIs for$\;\Delta {\bar{z}}_{m}$ and $\Delta {\bar{z}}_{f}$ using the posterior distributions of $\beta_{m}$,$\;\beta_{f}$,$\;G_{m}$,$\;G_{f}$ **,** andB produced by our animal model. In short, a single datapoint was selected at random from the posterior distribution for each parameter and used in equation (2) to calculate $\Delta {\bar{z}}_{m}$ and $\Delta {\bar{z}}_{f}$. This process was repeated for each datapoint in the posterior distribution (*n* = 4998) using the “MCMCglmm” package in R, and we used the posterior mean of these values as our parameter estimates of $\Delta {\bar{z}}_{m}$ and $\Delta {\bar{z}}_{f}$. To estimate the 95% CIs for $\Delta {\bar{z}}_{m}$ and $\Delta {\bar{z}}_{f}$, we used the HPD interval implemented in the *HPDinterval* function of the "MCMCglmm” package. The associated R code for this procedure is provided in the electronic Supporting Information (Appendix S5).

We examined the extent of ISC over the optimal regulation of P and C intake using a modified version of the rate of adaptation metric developed by Agrawal and Stinchcombe (2009). Although this metric was originally devised to examine the effects of genetic covariance on the response to selection within the sexes, it can easily be extended to quantify the effect that **B** has on the predicted evolutionary response of nutrient regulation in the sexes by measuring the following ratio:

(6) $$R\;=\;\frac{\Delta \bar{z}}{\Delta {\bar{z}}_{B\;=\;0}},$$

where $\Delta \bar{z}$ is the predicted response of P and C intake in the sexes estimated from equation (2) and $\Delta {\bar{z}}_{B\;=\;0}\;$are the same predicted responses when the genetic covariances in **B** have been set to zero. Therefore, ***R*** measures the predicted response of P and C intake in the sexes taking into account the covariances in **B** relative to response without these covariances (i.e., all traits are assumed to be genetically independent in the sexes). If ***R ***= 0.5, then the genetic covariance structure of **B** causes the response of regulated nutrient intake to increase only 50% as quickly as expected if these traits were genetically independent in the sexes. In contrast, ifR= 2.0, then the structure of **B** accelerates the response of regulated nutrient intake in the sexes twice as much as expected under genetic independence. If ***R ***= 1.0, then the structure of **B** has little effect on the response of regulated nutrient intake in the sexes (i.e., regulated nutrient intake is predicted to evolve as rapidly as if this trait was genetically independent in the sexes). To estimate the 95% CIs for ***R***, we used the approach outlined above for $\Delta {\bar{z}}_{m}$ and $\Delta {\bar{z}}_{f}$. The associated R code is provided in the electronic Supporting Information (Appendix S5).

### EXPERIMENT 3: SEX DIFFERENCES IN THE REGULATED INTAKE OF NUTRIENTS UNDER DIETARY CHOICE

To determine how male and female *T. commodus* regulate their intake of P and C under dietary choice, we conducted a second feeding choice experiment using outbred crickets taken at random from our culture. Nymphs were collected on the day they hatched and raised in groups of 50 individuals for three weeks before being separated and raised individually until eclosion to adulthood, following the procedure outlined in Experiment 2. At eclosion, 120 crickets of each sex were randomly divided between the same four diet pairs used in Experiment 2 (*n* = 30 crickets per diet pair for each sex). All experimental crickets were mated to a virgin cricket of the opposite sex (taken at random from the outbred culture) on the evening of day 8 post eclosion and this mating partner was removed the following morning. Experimental females were provided with a Petri dish of moist sand immediately after the mating partner was removed for oviposition. As in Experiment 2, diet consumption was measured every three days for a total of 21 days with fresh diet provided after each measurement period.

#### Statistical analysis

To determine if males and females showed a dietary preference within each diet pair, we used a paired *t*‐test comparing the total consumption of each diet in the pair. To examine sex differences in the regulated intake of P and C, we used a multivariate analysis of variance (MANOVA) that included diet pair and sex as main effects and P and C intake as the RVs. We followed our MANOVA model with univariate analyses of variance (ANOVAs) to determine which nutrient(s) contributed to any overall multivariate effects. As there were four diet pairs per sex, we used Fisher's least significant difference (LSD) post hoc analysis to determine which were significant at *P* < 0.05.

We calculated the RIP as the mean intake of P and C across the four diet pairs in each sex. To determine whether males and females optimally regulate their intake of nutrients under dietary choice with respect to LS, DRE, and LRE, we mapped the estimated RIP for male and female crickets onto their respective nutritional landscapes. We consider the RIP as being optimal if it coincides with the peak on the nutritional landscape. To test for differences in the RIP between the sexes, we used an analysis of covariance (ANCOVA) that included sex as a fixed effect, P intake, and the interaction between sex and P intake as a random effects and C intake as the RV. Significance of the interaction term indicates that the RIP differs significantly between the sexes. Our MANOVA and ANCOVA models were run using IBM® SPSS Statistics® software (IBM Corporation, version 23.0.0.0, Armonk, NY, USA).

## Results

### EXPERIMENT 1. THE EFFECT OF NUTRIENT INTAKE ON LIFE SPAN AND REPRODUCTIVE EFFORT IN THE SEXES

**Figure 1:**
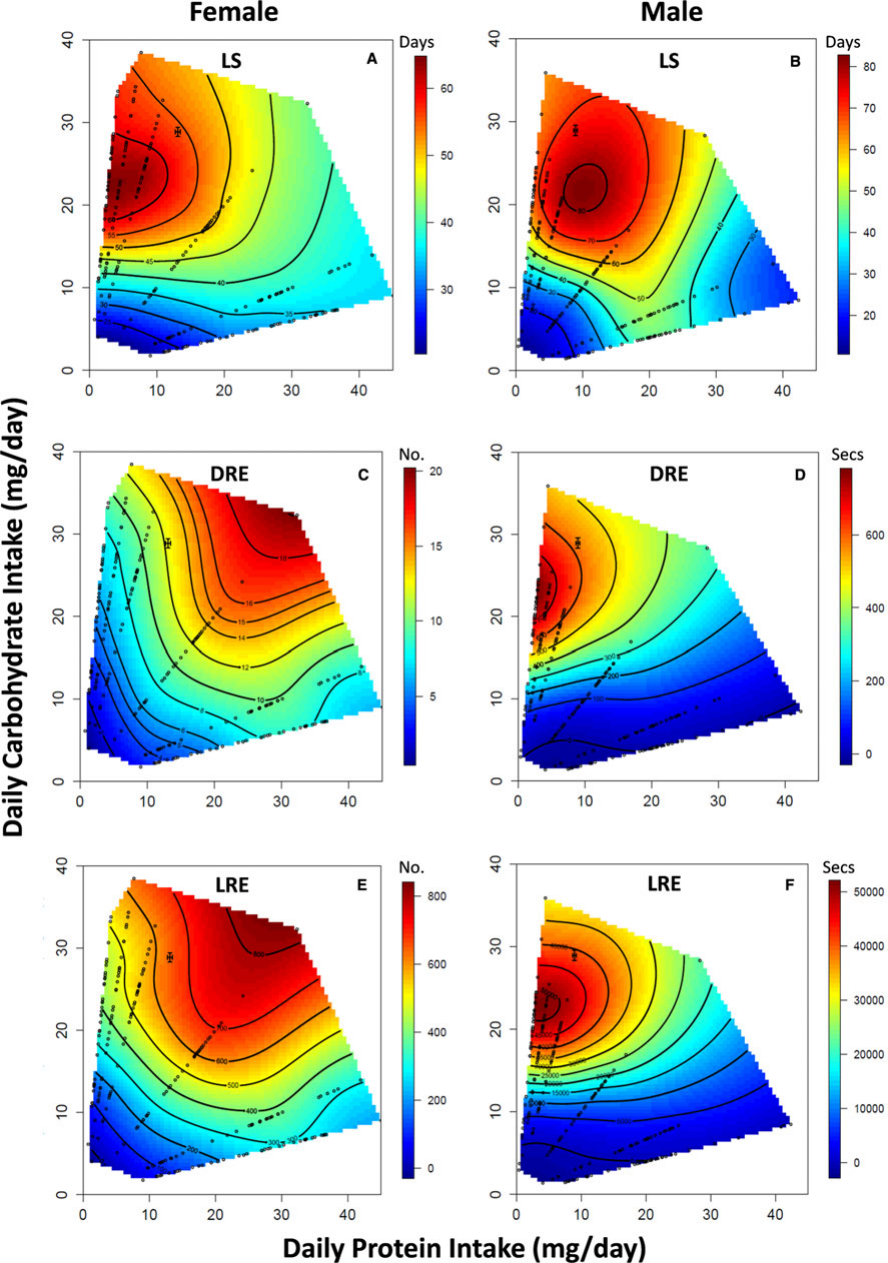
The nutritional landscapes for female and male life span (LS) (A and B, respectively), female and male daily reproductive effort (DRE) (C and D, respectively) and female and male lifetime reproductive effort (LRE) (E and F, respectively). In each landscape, the red regions represent higher values for the response variable, whereas blue regions represent lower values. The black cross in each figure represents the RIP (and 95% CIs) estimated in Experiment 3. The small black circles represent the actual feeding data for each cricket contained in the landscape. [Color figure can be viewed at wileyonlinelibrary.com]

Female LS increased linearly with C intake, but was not influenced by P intake (Table 1; Fig. 1A). The significant negative quadratic term for C intake suggests a peak in LS with the intake of this nutrient, and inspection of the nutritional landscape shows that this peak occurs at a high intake of C and a low intake of P in a P:C ratio of 1_P_:8_C_ (Table 1; Fig. 1A). There was also a significant negative correlational term providing further evidence that female LS was maximised at a high intake of C and a low intake of P (Table 1). In contrast, male LS increased linearly with the intake of both P and C, although it was more responsive to the intake of C than P (Table 1, Fig. 1B). The significant quadratic terms indicate a peak in male LS for both nutrients and inspection of the nutritional landscape shows that this peak occurs at a high intake of C and a low intake of P in a P:C ratio of 1_P_:2.5_C_ (Fig. 1B). There was also a significant negative correlational term providing further evidence that male LS is maximised at a high intake of C and low intake of P (Table 1). Formal statistical comparison of the nutritional landscapes using a sequential model approach showed that the linear and quadratic effects of P and C intake on LS in the sexes differed significantly, but the correlational effects of these nutrients did not (Table 2). The sex difference in linear effects was due to the fact that LS increased with P intake in males but not in females and also because LS is more responsive to C intake in males than females (Table 2). The sex difference in quadratic effects is due to the fact that there is a peak in LS with P intake in females but not in males (Table 2). However, the optima for LS in males and females appear to occur in similar regions of the nutritional landscapes (Fig. 1A and B), as evidenced by the relatively small angle between the two linear nutritional vectors (25.99°; Table 2). This suggests that despite the statistically significant sex differences in the effects of P and C on LS, these differences were relatively minor.

**Table 2:** Sequential model building approach showing differences in the linear and nonlinear effects of protein (P) and carbohydrate (C) ingestion on life span (LS), daily reproductive effort (DRE), and lifetime reproductive effort (LRE) between and within the sexes of *Teleogryllus commodus*. The angle (θ) with 95% CIs between the linear nutritional vectors for the response variables being compared is also provided. Letters provided next to *P* values indicate which nutrient(s) contributes to the overall significant difference (details are provided in the table footer)

	SS_R_	SS_C_	DF_1_	DF_2_	*F*	*P*	θ	95% CI
Females versus males								
LS								
Linear	253.60	243.44	2	424	8.85	0.0002^A^	25.99	13.11, 39.39
Quadratic	208.83	201.79	2	420	7.33	0.0007^B^		
Correlational	190.38	189.46	1	418	2.03	0.15		
DRE								
Linear	272.22	199.81	2	424	76.83	0.0001^C^	55.19	45.93, 64.40
Quadratic	187.97	168.05	2	420	24.89	0.0001^D^		
Correlational	167.64	158.03	1	418	25.41	0.0001		
LRE								
Linear	218.14	192.99	2	424	27.63	0.0001^E^	33.06	24.05, 42.42
Quadratic	177.74	165.68	2	420	15.29	0.0001^F^		
Correlational	163.86	160.27	1	418	9.35	0.002		
Females								
LS versus DRE								
Linear	325.66	282.74	2	438	118.45	0.0001^G^	51.18	38.14, 64.93
Quadratic	256.64	239.89	2	434	15.15	0.0001^H^		
Correlational	239.33	227.54	1	432	22.37	0.0001		
LS vs. LRE								
Linear	279.34	263.34	2	438	13.31	0.0001^I^	32.50	19.70, 45.66
Quadratic	235.88	224.64	2	434	20.52	0.0001^J^		
Correlational	221.67	215.61	1	432	12.15	0.0005		
DRE vs. LRE								
Linear	289.78	280.07	2	438	7.59	0.0006^K^	18.66	9.40, 28.65
Quadratic	225.82	225.09	2	434	0.70	0.50		
Correlational	222.17	221.23	1	432	1.84	0.18		
Males								
LS versus DRE								
Linear	180.69	160.51	2	410	25.77	0.0001^L^	30.48	21.65, 39.40
Quadratic	142.37	129.95	2	406	19.40	0.0001^M^		
Correlational	120.15	119.94	1	404	0.71	0.40		
LS versus LRE								
Linear	188.49	173.09	2	410	18.24	0.0001^N^	26.71	17.39, 35.97
Quadratic	151.81	142.83	2	406	12.76	0.0001^O^		
Correlational	134.19	134.12	1	404	0.21	0.65		
DRE versus LRE								
Linear	113.06	112.73	2	410	0.60	0.55	4.03	0.00, 10.69
Quadratic	109.02	108.63	2	406	0.72	0.48		
Correlational	97.11	97.07	1	404	0.17	0.68		

Univariate test:^A^P: *F*_1,424_ = 17.68, *P* = 0.0001, C: *F*_1,424_ = 3.98, *P* = 0.047; ^B^P × P: *F*_1,420_ = 12.39, *P* = 0.0005, C x C: *F*_1,420_ = 0.87, *P* = 0.35; ^C^ P: *F*_1,424_ = 83.05, *P* = 0.0001, C: *F*_1,424_ = 13.73, *P* = 0.0002; ^D^ P x P: *F*_1,420_ = 49.58, *P* = 0.0001, C x C: *F*_1,420_ = 0.43, *P* = 0.51; ^E^ P: *F*_1,424_ = 37.17, *P* = 0.0001, C: *F*_1,424_ = 1.53, *P* = 0.22; ^F^ P x P: *F*_1,420_ = 29.59, *P* = 0.0001, C x C: *F*_1,420_ = 3.37, *P* = 0.07;^G^ P: *F*_1,438_ = 48.92, *P* = 0.0001, C: *F*_1,438_ = 0.85, *P* = 0.36; ^H^ P x P: *F*_1,434_ = 22.66, *P* = 0.0001, C x C: *F*_1,434_ = 3.99, *P* = 0.046; ^I^ P: *F*_1,438_ = 25.67, *P* = 0.0001, C: *F*_1,438_ = 1.48, *P* = 0.22; ^J^ P x P: *F*_1,434_ = 18.26, *P* = 0.0001, C x C: *F*_1,434_ = 0.89, *P* = 0.35; ^K^ P: *F*_1,438_ = 4.47, *P* = 0.035, C: *F*_1,438_ = 4.43, *P* = 0.036; ^L^ P: *F*_1,410_ = 38.16, *P* = 0.0001, C: *F*_1,410_ = 0.33, *P* = 0.57; ^M^ P x P: *F*_1,406_ = 36.17, *P* = 0.0001, C x C: *F*_1,406_ = 4.62, *P* = 0.03; ^N^ P: *F*_1,410_ = 27.15, *P* = 0.0001, C: *F*_1,410_ = 0.21, *P* = 0.65; ^O^ P x P = *F*_1,406_ = 22.28, *P* = 0.0001, C x C: *F*_1,406_ = 4.91, *P* = 0.03.

**Figure 2:**
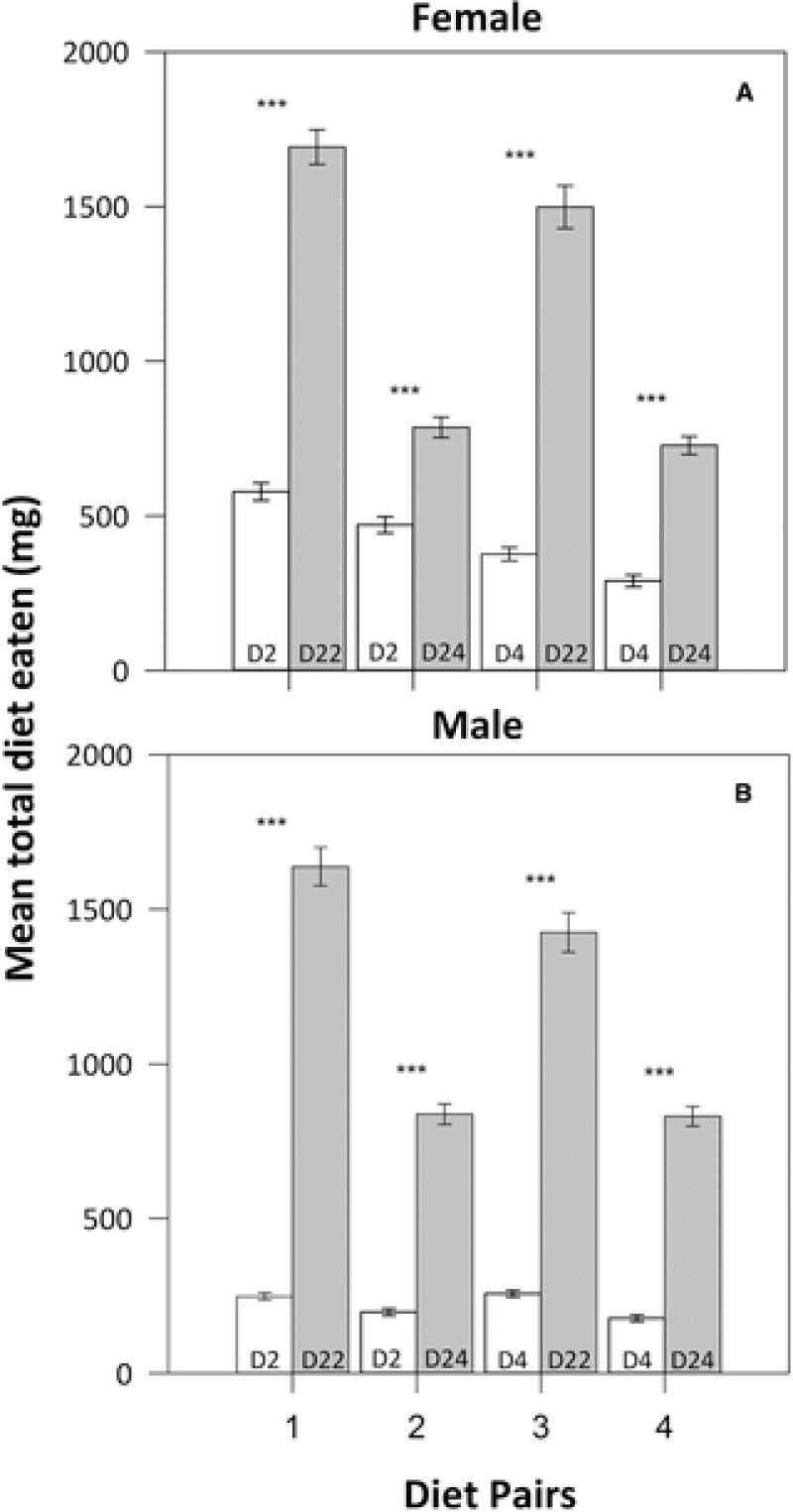
The mean (and 95% CIs) consumption from each diet by female (A) and male (B) *Teleogryllus commodus* in each of the four diet pairs in Experiment 3. In each diet pair, the gray column represents the diet higher in carbohydrate content and the white column the diet higher in protein content. The number of each diet is provided at the base of each column. The asterisk provided above the bars in each diet pair indicates that mean consumption differs significantly at *P* < 0.05 as determined with a paired *t*‐test.

Female DRE increased linearly with the intake of P and C, with both nutrients having an almost equal effect on this trait (Table 1). There was also a significant negative quadratic term for P intake indicating a peak in DRE with the intake of this nutrient. Inspection of the nutritional landscape shows that this peak occurs at high intakes of P and C in a P:C ratio of 1_P_:1_C_ (Fig. 1C). The significant positive correlational term further demonstrates that DRE increased with the intake of both nutrients in females (Fig. 2C; Table 1). In contrast, male DRE increased linearly with the intake of C and significantly decreased with the intake of P (Table 1). The significant negative quadratic term for C intake indicates a peak in DRE with the intake of this nutrient and inspection of the nutritional landscape shows that this peak occurs at a high intake of C and a low intake of P at a P:C ratio of 1_P_:8_C_ (Fig. 2D; Table 1). The significant negative correlational term further demonstrates that DRE in males is maximized at a high intake of C and low intake of P (Fig. 2D; Table 1). Formal statistical comparison of the landscapes showed that the linear, quadratic, and correlational effects of P and C intake on DRE differed significantly in the sexes (Table 2). The sex differences in the linear effects is due to the fact that DRE increases with P intake in females but decreases with P intake in males and also because DRE is more responsive to the intake of C in males than females (Table 2). The sex differences in the quadratic effects is due to the fact that DRE peaks with P intake in females but not in males, whereas the sex differences in the correlational effects occur because the covariance between nutrients has a positive effect on DRE in females, but a negative effect in males (Table 2). The large angle (55.19°) between the linear nutritional vectors indicates that the optima for DRE occur in different regions of the nutritional landscape for males and females (Table 2; Fig. 1C and D).

The effects of P and C intake on LRE in the sexes show a similar pattern to that shown for DRE. LRE in females increased linearly with the intake of both P and C, although this trait was more responsive to the intake of C than P (Table 1). There were significant negative quadratic terms for both nutrients indicating a peak in LRE with both nutrients, and inspection of the nutritional landscape shows that this peak occurs at a high intake of P and C in a P:C ratio of 1_P_:1.5_C_ (Fig. 2E; Table 1). In contrast, LRE in males increased linearly with C intake and decreased linearly with P intake (Table 1). The significant negative quadratic term for C intake indicates a peak in LRE with the intake of this nutrient, and inspection of the nutritional landscape shows that this peak occurs at a high intake of C and a low intake of P at a P:C ratio of 1_P_:8_C_ (Fig. 2F; Table 1). There was also a significant negative correlational gradient, which further confirms that LRE is optimized at a high intake of C and a low intake of P (Fig. 2F; Table 1). Formal statistical comparison of the landscapes showed that the linear, quadratic, and correlational effects of P and C intake on LRE differed significantly in the sexes (Table 2). The sex differences in the linear effects are due to the fact that DRE increases with P intake in females, but decreases with P intake in males (Table 2). The sex differences in the quadratic effects reflects that LRE peaks with P intake in females but not in males, whereas the sex differences in the correlational effects occur because the covariance between nutrients has a negative effect on LRE in males but not in females (Table 2). The optima for LRE in males and females occur in different regions on the nutritional landscapes (Fig. 1E and F), as evidenced by the relatively large angle between the two linear nutritional vectors (33.06°; Table 2).

The nutritional landscapes for LS, DRE, and LRE also differed within each sex (Table 2). In females, the linear, quadratic and correlational effects of P and C intake on LS differed significantly from the effects of these nutrients on DRE and LRE (Table 2). In both cases, the difference in linear effects was due to the positive effect of P on DRE and LRE but not on LS (Table 2). The difference in quadratic effects was due to the fact that DRE and LRE peaked with P intake but LS did not and because the peak in LS with C intake was more pronounced than the peaks for DRE and LRE (Table 2). The difference in correlational effects arose because the covariance between P and C intake had a negative effect on LS, a positive effect on DRE and had no effect on LRE (Table 2). There was also a significant difference in the linear effects of P and C intake on DRE and LRE, driven by DRE being more responsive than LRE to P intake, whereas the reverse pattern was true for C intake (Table 2). Collectively, this suggests that LS peaks in a different region on the nutritional landscape than DRE and LRE (Fig. 1A, C, and E), a finding supported by the large angles between the nutritional vectors for these traits (51.18° and 32.50°, respectively; Table 2). In contrast, DRE and LRE peak in similar regions of the landscape (Fig. 1C and E), as indicated by the much smaller angle between the nutritional vectors for these traits (18.66°, Table 2). As LS cannot be optimized at the same intake of nutrients that maximizes DRE and LRE (and vice versa), this indicates a trade‐off between these traits in females.

In contrast, only the linear and quadratic effects of P and C intake on LS differed significantly from the effects of these nutrients on DRE and LRE in males (Table 2). In both instances, the difference in linear effects was the result of P intake having a positive effect on LS but a negative effect on DRE and LRE (Table 2). Furthermore, the difference in quadratic effects was due to the fact that LS peaked with P intake but DRE and LRE did not and because the peak in LS with C intake was more pronounced than the peaks in DRE and LRE with the intake of this nutrient (Table 2). In contrast to females, the linear, quadratic and correlational effects of nutrient intake on DRE and LRE in males did not differ significantly, indicating that these traits peak in the same region in nutrient space, as demonstrated by the small angle between the linear nutritional vectors for these traits (4.03°, Table 2). Collectively, this suggests that the nutritional optimum for LS in males occurs in a different region than the optima for DRE and LRE (Fig. 1B, D, and E). However, the angle between the nutritional linear vector for LS and DRE (30.48°) and LS and LRE (26.71°) is smaller than observed in females (51.18° and 32.50°, respectively), suggesting that while these traits trade‐off in males, the strength of this trade‐off is weaker than in females (Table 2).

### EXPERIMENT 2. THE QUANTITATIVE GENETICS OF NUTRIENT REGULATION

The **G** matrix for the regulated intake of P and C in male and female *T. commodus* is presented in Table 3. The regulated intake of P and C was significantly heritable in both sexes and heritability estimates are of a similar magnitude in each sex (Table 3). However, in both sexes, the heritability estimates for the regulated intake of P were over twice as large as those for the regulated intake of C (Table 3). Furthermore, the genetic correlations between the regulated intake of P and C was significant and positive within both sexes (*r*_M_ and *r*_F_), although the estimate was higher for males than females (Table 3). Most importantly, we show strong and significant positive genetic correlations (*r*_MF_) for the regulated intake of P and C between the sexes (Table 3). Together with the sex differences in the effects of P and C on LS, DRE, and LRE we document in Experiment 1, these strong estimates of *r*_MF_ demonstrate the potential for ISC to constrain the evolution of nutrient regulation in *T. commodus*.

**Table 3:** Additive genetic variance–covariance (**G**) matrix for the regulated intake of protein (P) and carbohydrate (C) in male and female *Teleogryllus commodus*

	P_m_	C_m_	P_f_	C_f_
P_m_	0.34	*0.79*	*0.79*	*0.46*
	(0.22, 0.49)	*(0.60, 0.88)*	*(0.65, 0.89)*	*(0.22, 0.70)*
C_m_	0.18	0.20	*0.74*	*0.55*
	(0.12, 0.30)	(0.13, 0.31)	*(0.54, 0.85)*	*(0.25, 0.73)*
P_f_	0.24	0.17	0.31	*0.60*
	(0.15, 0.38)	(0.10, 0.28)	(0.20, 0.45)	*(0.36, 0.78)*
C_f_	0.11	0.09	0.13	0.15
	(0.04, 0.20)	(0.04, 0.17)	(0.06, 0.22)	(0.10, 0.26)

The subscripts *m* and *f* refer to males and females, respectively. The additive genetic variance within males and females is provided along the diagonal. As our analyses were performed on standardized nutrient intake, our estimates of additive variance are equivalent to heritabilities (*h* ^2^). The additive genetic covariance within and between the sexes is provided below the diagonal and additive genetic correlations (*r*_M,_ *r*_F_, and *r*_MF_) are provided in italics above the diagonal. The 95% CIs are provided beneath each estimate in brackets.

Our estimates of$\;\Delta \bar{z}$, $\Delta {\bar{z}}_{B\;=\;0}$, and ***R*** for LS, DRE, and LRE in the sexes, as well as their associated 95% CIs, are provided in Table 4. With the exception of the regulated intake of C for female DRE, estimates of $\Delta \bar{z}$ exceeded those for $\Delta {\bar{z}}_{B\;=\;0}$ for all other traits in the sexes (Table 4). In males, ***R*** values for the regulated intake of P and C for DRE and the regulated intake of C for LRE were significantly greater than 1.0 (i.e., 95% CIs did not overlap 1.0), whereas the remaining traits did not differ significantly from 1.0 (Table 4). In females, the ***R*** value for the regulated intake of P for LS was significantly greater than 1.0, however, all other traits did not differ significantly from 1.0 (Table 4). Collectively, this suggests that for most traits in both sexes, **B** did little to alter the predicted evolutionary response of the regulated intake of nutrients and, in those few instances where it did, **B** appeared to accelerate the predicted response rather than constrain it.

**Table 4:** The predicted evolutionary response of the regulated intake of protein (P) and carbohydrate (C) in the sexes of *Teleogryllus commodus* when B is estimated directly from our breeding design ($\Delta \bar{z}$) versus when it has been set to zero ($\Delta {\bar{z}}_{B\;=\;0}$).We also provide the corresponding *R* genetic constraint metric of Agrawal & Stinchcombe (2009). The 95% CIs are provided in brackets beneath each estimate. Values in bold are considered significantly greater than 1.0, as the 95% CIs do not overlap this value

	Males			Females		
	$\Delta \bar{z}$	$\Delta {\bar{z}}_{B\;=\;0}$	*R*	$\Delta \bar{z}$	$\Delta {\bar{z}}_{B\;=\;0}$	*R*
LS						
P	0.15	0.11	1.37	0.13	0.03	3.82
	(0.09, 0.23)	(0.07, 0.15)	(0.65, 2.40)	(0.08, 0.21)	(0.005, 0.07)	(1.01, 13.01)
C	0.13	0.09	1.42	0.10	0.05	2.00
	(0.07, 0.20)	(0.06, 0.13)	(0.66, 2.38)	(0.05, 0.15)	(0.02, 0.08)	(0.72, 3.91)
DRE						
P	0.15	0.05	3.43	0.17	0.13	1.37
	(0.09, 0.23)	(0.02, 0.07)	(1.31, 6.92)	(0.10, 0.25)	(0.08, 0.18)	(0.69, 2.39)
C	0.14	0.06	2.32	0.11	0.13	0.83
	(0.09, 0.21)	(0.03, 0.09)	(1.15, 4.19)	(0.05, 0.17)	(0.09, 0.18)	(0.39, 1.42)
LRE						
P	0.15	0.05	2.88	0.16	0.11	1.45
	(0.08, 0.23)	(0.03, 0.08)	(1.18, 5.18)	(0.09, 0.24)	(0.06, 0.16)	(0.70, 2.59)
C	0.14	0.06	2.11	0.11	0.09	1.28
	(0.08, 0.21)	(0.04, 0.09)	(0.92, 3.75)	(0.06, 0.18)	(0.05, 0.13)	(0.54, 2.34)

We also provide the corresponding *R* genetic constraint metric of Agrawal and Stinchcombe (2009). The 95% CIs are provided in brackets beneath each estimate. Values in bold are considered significantly greater than 1.0, as the 95% CIs do not overlap this value.

### EXPERIMENT 3. SEX DIFFERENCES IN THE REGULATED INTAKE OF NUTRIENTS UNDER DIETARY CHOICE

**Figure 3:**
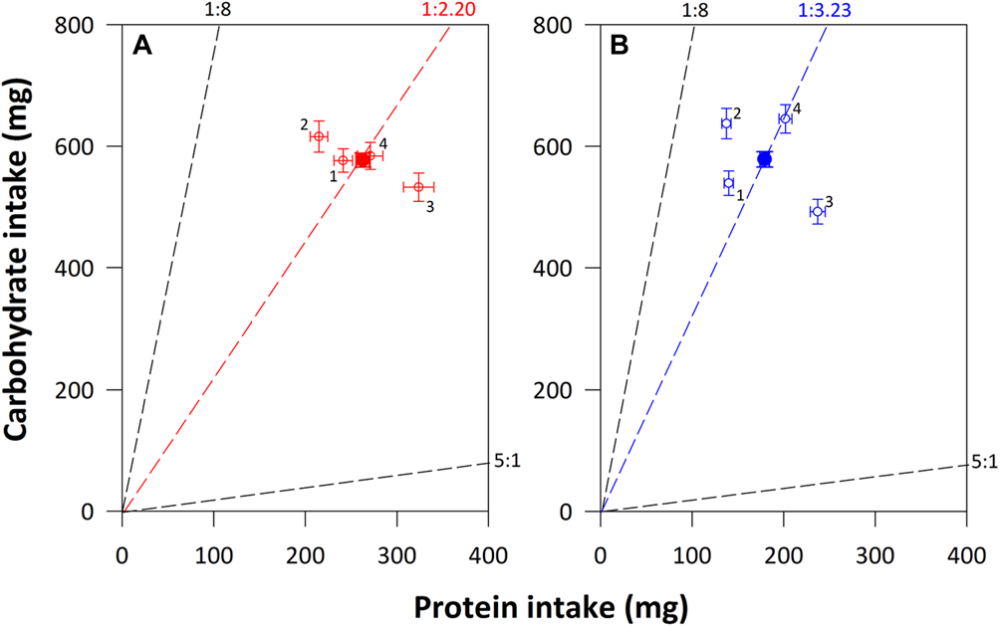
The mean (and 95% CIs) protein (P) and carbohydrate (C) intake of female (A, red symbols) and male (B, blue symbols) *Teleogryllus commodus* on the four different diet pairs contained in Experiment 3 (open symbols, labeled with diet pair number). The mean (and 95% CIs) intake of P and C across these diet pairs, known as the regulated intake point (RIP), is provided for females (closed red symbol) and males (closed blue symbol), as well as the P:C ratio corresponding to the RIP (red and blue dashed line, respectively). The black dashed lines in each figure (at P:C ratios of 5:1 and 1:8) represent the outer nutritional rails for the individual diets contained in the diet pairs. Consequently, crickets are able to feed to any position within these rails by exerting dietary choice. [Color figure can be viewed at wileyonlinelibrary.com]

Males and females both showed a clear preference for the high C diet over the high P diet in each diet pair (Fig. 2). A MANOVA revealed a significant multivariate effect of sex and diet pair, but not a significant interaction between sex and diet pair, on the intake of nutrients under dietary choice (Table 5). Univariate ANOVAs revealed that P intake but not C intake contributed to the observed difference between the sexes and that both nutrients contributed to the observed difference across diet pairs (Table 5). In females, post hoc analysis showed that the pattern of P intake was diet pair 2 < 1 < 4 < 3 and C intake was diet pair 3 < 1 = 4 = 2 (Fig. 3A), whereas in males the pattern of P intake was diet pair 2 = 1 < 4 < 3 and C intake was diet pair 3 < 1 < 2 = 4 (Fig. 3B).

**Table 5:** Multivariate analysis of variance (MANOVA) examining the effects of sex and diet pair on the total intake of protein (P) and carbohydrates (C) in *Teleogryllus commodus*

	MANOVA			
Model terms	Pillai's trace	Df	*F*	*P*
Sex (A)	0.46	2,231	96.91	0.0001
Diet pair (B)	0.60	6,464	33.44	0.0001
A x B	0.03	6,464	1.31	0.25

	Univariate ANOVAs			
Model terms	Nutrient	df	*F*	*P*
Sex (A)	P	1,232	135.91	0.0001
	C	1,232	0.01	0.93
Diet pair (B)	P	3,232	43.33	0.0001
	C	3,232	11.06	0.0001
A x B	P	3,232	0.93	0.43
	C	3,232	2.37	0.07

This overall multivariate model was followed by a series of univariate ANOVAs to determine which nutrients contributed to any overall multivariate effects and Fisher's LSD post hoc analysis to determine the order.

The RIP was at a mean P intake of 262.69 ± 7.33 mg and C intake of 577.10 ± 11.54 mg for females (1_P_:2.20_C_), a mean P intake of 179.22 ± 5.02 mg and C intake of 578.57 ± 12.51 mg for males (1_P_:3.23_C_) and can be visualized in Figure 3. There was a significant difference between the sexes in the RIP, with females choosing to consume a more P‐biased diet than males (sex: *F*_1,236_ = 4.08, *P* = 0.04, P intake: *F*_1,236_ = 13.96, *P* = 0.0001, sex by P intake: *F*_1,236_ = 4.96, *P* = 0.027; Fig. 3). Mapping the RIP for males and females onto the nutritional landscapes presented in Figure 1 showed that the RIP did not coincide exactly with the peaks for LS, DRE, or LRE, suggesting that neither sex are optimally regulating their intake of P and C under dietary choice with regard to these traits.

## Discussion

Sex differences in LS are taxonomically widespread in the animal kingdom and understanding the evolutionary and mechanistic factors driving this divergence is a central challenge to evolutionary biologists and biogerontologists (Maklakov and Lummaa 2013; Austad and Fischer 2016). Recently, IASC over the optimal intake of nutrients for LS and reproduction has been proposed as a potential mechanism explaining this pattern (Maklakov et al. 2008; Maklakov and Lummaa 2013; Jensen et al. 2015). This argument is based on the fact that the intake of various key nutrients are known to have a profound effect on LS and reproduction in a wide range of species (e.g., Lee et al. 2008; Fanson et al. 2009; Hawley et al. 2015; Rho and Lee 2016) and in many instances, these nutrients have contrasting effects on LS and reproduction in the sexes (e.g., Maklakov et al. 2008; Reddiex et al. 2013; Harrison et al. 2014; Jensen et al. 2015). If nutrient intake has a common genetic basis in the sexes, this will generate IASC over the optimal intake of nutrients that can prevent one or both sexes from evolving to their sex‐specific nutritional optima for LS or reproduction by constraining the evolution of sexual dimorphism in nutrient regulation. In this study, we integrated the Geometric Framework with quantitative genetics to examine the importance of IASC over the optimal intake of P and C in the black field cricket, *T. commodus*. We show significant sex differences in the effects of P and C intake on LS, DRE, and LRE and that there are strong positive additive genetic correlations between the sexes for the regulated intake of these nutrients under dietary choice. Although this is often used to formally document the existence of IASC (Bonduriansky and Chenoweth 2009), we show that the between sex additive genetic covariance matrix (**B**) had very little effect on the predicted response of nutrient regulation in the sexes and where an effect was detected, the structure of **B** actually accelerated this predicted response. Therefore, contrary to previous work on this species (Maklakov et al. 2008), our work suggests that IASC over the optimal intake of nutrients for LS, DRE, and LRE is likely to be weak in *T. commodus*. In agreement with this view, we found clear sexual dimorphism in the regulated intake of P and C under dietary choice, with females regulating their intake of these nutrients to a higher P:C ratio than males. However, nutrient regulation did not coincide with the nutritional optima for LS, DRE, or LRE in males or females, suggesting that IASC is either not completely resolved in this species or that other possible constraints exist.

Our study shows that P and C intake had clear effects on LS in male and female *T. commodus*. In males, LS was maximized at a high intake of nutrients (i.e., high calories) in a P:C ratio of 1_P_:2.5_C_ (Fig. 1B), whereas female LS was also maximized at a high intake of nutrients but in a P:C ratio of 1_P_:8_C_ (Fig. 1A). Although there were statistically significant sex differences in the effects of P and C on LS, these differences were relatively minor and the small angle (25.99°) between the linear nutritional vectors for LS in the sexes indicates that LS is maximized in similar regions of nutrient space in the sexes (Fig. 1A and B). This finding is largely consistent with earlier work on this species that also found relatively minor differences in the effects of P and C intake on LS between the sexes, with LS being maximized at a P:C ratio of 1_P_:5_C_ and 1_P_:8_C_ in males and females, respectively (Maklakov et al. 2008). Moreover, our general finding that LS is extended at a high intake of diets with low P:C ratio is also broadly consistent with a large number of Geometric Framework studies on invertebrates (e.g., Lee et al. 2008; Fanson et al. 2009; Fanson and Taylor 2012; Harrison et al. 2014; Hawley et al. 2015; Jensen et al. 2015; Rho and Lee 2016), as well as in mice (Solon‐Biet et al. 2014). Our work therefore adds to this already extensive list of studies directly challenging a central role for caloric restriction in extending LS.

In contrast to LS, there was a much larger difference in the effects of P and C intake on DRE, and to a lesser extent LRE, between the sexes. For males, DRE and LRE were both maximized at a high intake of nutrients in a P:C ratio of 1_P_:8_C_ (Fig. 1D and F), whereas DRE and LRE in females were maximized at a high intake of nutrients in a P:C ratio of 1_P_:1_C_ (Fig. 1C) and 1_P_:1.5_C_ (Fig. 1E), respectively. For DRE, the major difference between the sexes occurred because of the contrasting effects of P intake (daily egg production increased but nightly calling effort decreased with P intake) and also because male DRE was more responsive to the intake of C than females. Consequently, DRE was maximized in different regions of the nutritional landscape in males and females (Fig. 1C and D), as illustrated by the large angle between the linear nutritional vectors for this trait in the sexes (55.19°). The divergence between the sexes for the effects of nutrient intake on LRE was less pronounced, with the angle of 33.06° between the linear nutritional vectors in the sexes, due to the fact that P intake does not influence LS in females (Table 1). This pattern of nutrient effects on DRE and LRE in the sexes largely mirrors the findings of Maklakov et al. (2008) who also found that these traits were maximized at a high intake of nutrients in a P:C ratio of 1_P_:8_C_ and 1_P_:1_C_ in males and females, respectively. It has been argued that these differences in the optimal nutritional requirements for reproduction reflect the divergent reproductive strategies of the sexes.

In most species, males compete for access to females and those with the most elaborate sexual trait or behavior are the most successful (Bonduriansky et al. 2008). In *T. commodus*, a key determinant of male mating success is the production of an acoustic advertisement call (Bentsen et al. 2006). To fuel the production of this metabolically costly signal (Kavanagh 1987), males require a high intake of C to provide an abundant source of energy that can be easily and rapidly accessed after digestion (Maklakov et al. 2008; South et al. 2011). In contrast, females typically do not have to compete for matings and their reproductive success is determined by the number of eggs they produce rather than the number of matings they achieve (Bonduriansky et al. 2008). In many insect species, the consumption of P stimulates oogenesis and regulates vitellogenesis (Wheeler 1996). It is therefore, not surprising that a high intake of P is required in females to maximize egg production in most species examined (Lee et al. 2008; Maklakov et al. 2008; Fanson et al. 2009; Fanson and Taylor 2012; Reddiex et al. 2013; Jensen et al. 2015; but see Bunning et al. 2016) and this requirement is typically much greater than that of males (Reddiex et al. 2013; Harrison et al. 2014; Jensen et al. 2015).

The trade‐off between LS and reproduction is a key feature of many evolutionary theories of ageing (e.g., Williams 1966; Barnes and Partridge 2003) and is based on the argument that both life‐history traits cannot be maximized simultaneously because increasing reproductive effort diverts essential resources away from somatic maintenance and LS (Van Noordwijk and de Jong 1986). The intake of calories has traditionally been viewed as the limiting resource that regulates this trade‐off (e.g., Gadgil and Bossert 1970). Our work shows, however, that the trade‐off between LS and reproduction in *T. commodus* is based on the intake of P and C and not calories per se and is more pronounced in females than males. In females, LS and DRE were maximized in very different regions on the nutritional landscape (Fig. 1A and C), as illustrated by the large angle (51.18°) between the linear nutritional vectors for these traits, meaning that these traits cannot be maximized at the same intake of P and C. In contrast, the smaller angle (30.48°) between the linear nutritional vectors and the much broader nutritional optima for DRE and LS in males (Fig. 1B and D) means that there are certain intakes of P and C where both traits can be maximized. This observed sex difference in the strength of the trade‐off between reproduction and LS appears widespread in the animal kingdom and supports the view that reproduction is more costly in females than males (e.g., Bonduriansky et al. 2008; Hayward and Gillooly 2011). A similar pattern was also observed for LS and LRE in the sexes, although it was far less pronounced than the trade‐offs between LS and DRE in both females and males, with the angles between the linear nutritional vectors being 32.50° and 26.71°, respectively. This suggests that LS has a greater effect on LRE in females than males and is supported by the fact that the linear effects of P and C intake on DRE and LRE differ significantly in females but not males (Table 2), and that the angle between the nutritional vectors for these traits is much larger in females (18.66°) than males (4.03°). This finding is in general agreement with sexual selection theory, which predicts that female fitness will be maximized through a “low wear and tear” strategy that yields moderate rates of return over longer time periods (Graves 2007; Bonduriansky et al. 2008), and is consistent with the fact that female *T. commodus*, on average, live longer than males under both natural (Zajitschek et al. 2009a) and seminatural (Zajitschek et al. 2009b) conditions.

Understanding the relative contribution of genes and the environment to food selection remains a major challenge in the fields of nutrigenetics and nutrigenomics (e.g., Reed 2008; Liu et al. 2013). There is convincing support from studies on humans and rodent models that macronutrient intake has a genetic basis. For example, twin studies in humans have shown that the intake of protein, carbohydrates, and fat all have a genetic basis, although considerable variation in genetic estimates for different nationalities and the sexes were found (Liu et al. 2013). Far less, however, is known about the genetics of macronutrient intake in insects, with only a single study examining the genetics of P and C intake under dietary choice in male and female *D. melanogaster* (Reddiex et al. 2013). This study found that heritability estimates for the intake of both nutrients were higher in females than males and were generally lower for P than C intake (Reddiex et al. 2013). Moreover, genetic correlations between P and C intake were positive and of similar magnitude in the sexes, and while there was a strong a positive intersexual genetic correlation for C intake, there was only a weak genetic correlation between the sexes for P intake. A limitation of this study, however, is that nutrient preference was restricted to only two diets (pure yeast and sucrose solutions at 30 g per 100 mL) over a four‐day period and may therefore not provide an accurate picture of how individuals regulate their intake of P and C. In contrast, as we present each genotype with four different diet pairs over 21 days, our study not only covers a much broader nutritional range for a longer time period, but also provides genetic estimates for the RIP for P and C in the sexes (as opposed to nutrient preference). We show that the regulated intake of P and C intake in *T. commodus* is heritable and of similar magnitude in the sexes, but our *h* ^2^ estimates for P intake were over twice as large as for C intake (Table 3). Furthermore, we found a stronger positive genetic correlation between the regulated intake of P and C in males than females and a stronger positive intersexual genetic correlation for the regulated intake of P than C (Table 3). This indicates that the regulation of P intake is under stronger genetic control than the regulation of C intake in *T. commodus*, and that the regulated intake of both nutrients is unlikely to evolve independently within and between the sexes. Such dominant regulation of P intake over C and lipid intake appears widespread in animals (Raubenheimer et al., 2015) and it has been argued that when P is in limited supply, this regulation can increase dietary consumption and the overall intake of energy leading to obesity and other metabolic disorders (referred to as the “protein leverage hypothesis”; Simpson and Raubenheimer 2005). Although there is considerable support for this hypothesis at the phenotypic level (e.g., Gosby et al. 2014), our work provides novel insight into how this hypothesis can function at the genetic level.

IASC reflects a fundamental conflict between the shared and divergent aspects of the biology of the sexes (Bonduriansky and Chenoweth 2009) and is now accepted as a key evolutionary process with widespread implications, including the preservation of genetic variation (e.g., Prasad et al. 2006), diminishing the benefits of sexual selection (e.g., Brommer et al. 2007), increasing the risk of extinction (e.g., Kokko and Brooks 2003) and driving the speciation process (e.g., Gavrilets and Hayashi 2005). IASC arises whenever traits shared by the sexes have a common genetic basis but are subject to contrasting selection (Bonduriansky and Chenoweth 2009). Consequently, our finding that there are sex differences in the effects of P and C intake on LS, DRE, and LRE and positive intersexual genetic correlations for the regulated intake of P and C provides the conditions necessary for IASC over the optimal intake of these nutrients for LS, DRE, and LRE to operate in *T. commodus*. Our work cautions against this approach, however, by showing that the between sex additive genetic covariance matrix (**B**) did little to constrain the predicted evolutionary response of nutrient regulation in the sexes. In fact, in all instances where **B** was shown to significantly influence the predicted evolutionary response of nutrient regulation compared to when **B** was set to zero, it accelerated the predicted evolutionary response rather than constrained it (i.e., $\Delta \bar{z}$ > $\Delta {\bar{z}}_{B\;=\;0}$; Table 4) (Agrawal and Stinchombe 2009). This suggests that either the divergent effects of P and C intake on LS, DRE, and LRE in the sexes and/or the architecture of **B** is not sufficient for IASC to act as a major evolutionary constraint on nutrient regulation in *T. commodus*. In theory, phenotypic traits will be free to evolve independently in the sexes when *r*_MF_ is 0 but will be most constrained when *r*_MF_ is 1 (Lande 1980). Although our estimates of *r*_MF_ for the regulated intake of P and C were significantly lower than 1, they were still high and significantly greater than 0 (Table 3), indicating that the evolution of nutrient regulation is unlikely to occur independently in the sexes. It is, therefore, more likely that the lack of strong IASC over the optimal intake of nutrients for LS, DRE, and LRE we observe in *T. commodus* reflects the relatively small divergence in the effects of P and C intake on these traits in the sexes; a conclusion that was also reached by Reddiex et al. (2013) for *D. melanogaster*. This point is best illustrated by comparison to a study on the Indian meal moth (*Plodia interpunctella*) that documented strong IASC over life‐history traits (development time, body size, and LS) (Lewis et al. 2011). With the exception of *r*_MF_ for development time and the heritability of this trait in males, which are high because this trait is sex linked in *P. interpunctella*, all other estimates in **B** were considerably weaker than documented in our study. Selection on these life‐history traits, however, was almost directly opposing in the sexes, with much larger angle (127.91°) between the linear selection gradients than reported for LS (25.99°), DRE (55.19°), or LRE (33.06°) in our study.

Optimal foraging theory predicts that individuals will evolve foraging strategies to maximize their fitness (Stephens and Krebs 1986). Traditionally, it was argued that this was primarily achieved by maximizing the rate of energy intake (Stephens and Krebs 1986), but more recent studies have shown that individuals can also optimally regulate their intake of specific nutrients (e.g., Simpson et al. 2004; Jensen et al. 2012). Our finding that P and C intake has divergent effects on LS, DRE, and LRE in the sexes demonstrates that there is a clear benefit to male and female *T. commodus* of independently regulating their intake of these nutrients. Indeed, we show that *T. commodus* exhibits clear sexual dimorphism in the regulated intake of P and C under dietary choice, with females regulating to a higher intake of P than males (1_P_:3.23_C_ in males and 1_P_:2.20_C_ in females), which aligns with their increased requirement for this nutrient to maximize egg production. An identical pattern was shown in the cricket *G. veletis* (Harrison et al. 2014) where the sexes show similar divergence in the effects of P and C intake on LS and reproduction and females regulate to a higher intake of P than males when given dietary choice (1_P_:4.1_C_ in males and 1.3_P_:3_C_ in females). Similarly, a recent study on the beetle *Tenebrio molitor* (Rho and Lee 2016) also found that females regulate to a higher intake of P than males, although whether these nutrients have divergent effects on reproduction in the sexes was not explicitly tested. In contrast, initial work on *T. commodus* (Maklakov et al. 2008), as well as a more recent study on *D. melanogaster* (Jensen et al. 2015), showed clear sex differences in the effects of P and C on LS and reproduction but found that males and females regulate their intake of nutrients to a common P:C ratio (1_P_:2.96_C_ in *T. commodus* and 1_P_:4_C_ in *D. melanogaster*). It was argued in both of these studies that this shared pattern of nutrient regulation was preventing the sexes from reaching their sex‐specific nutritional optima for LS and reproduction and was, therefore, a likely signal of the presence of IASC over the optimal intake of nutrients (Maklakov et al. 2008; Jensen et al. 2015). Our finding that IASC over the optimal intake of P and C for LS, DRE, and LRE is weak and that the sexes show a clear dimorphism in nutrient regulation, therefore suggests that IASC may be resolved in *T. commodus* or at least be in the initial stages of resolution. Given sufficient evolutionary time, selection is expected to resolve IASC and a variety of mechanisms are known to facilitate this process (e.g., sex‐linked modifiers, gene duplication, sex‐biased gene expression, sex linkage, and genomic imprinting) (Bonduriansky and Chenoweth 2009). However, our finding that the RIP for both males and females did not coincide perfectly with the respective optima for LS, DRE, or LRE (Fig. 1) does suggest that IASC over the optimal intake of nutrients is unlikely to be fully resolved in this species. The extent to which IASC has indeed been revolved, and the proximate mechanisms that may be responsible in *T. commodus* clearly requires further study. It is likely that using genomic approaches to probe the genetic mechanisms known to resolve IASC will prove fruitful in this endeavor (Bonduriansky and Chenoweth 2009).

It is possible that processes other than IASC may also be preventing male and female *T. commodus* from optimally regulating their intake of nutrients for LS, DRE, and LRE. One possibility is that males and females are regulating their intake of nutrients to maximize the expression of other more heavily prioritized traits. Although lifetime egg production is likely to provide a good measure of fitness in females, the same is unlikely to be true for lifetime calling effort in males. This is because a myriad of other traits, such as the production of sperm, other sexual signals (e.g., cuticular hydrocarbons), and the ability to sire offspring under competition, are also likely to be important components of fitness. Although the effects of P and C intake on these traits is not known in *T. commodus*, studies on other insect species have shown that sperm production (1_P_:2_C_, Bunning et al. 2015) and cuticular hydrocarbons expression (1_P_:1.5_C_, Rapkin et al. 2016) are maximized at lower P:C ratio than the RIP in male *T. commodus*, whereas competitive mating success (1_P_:16_C_, Jensen et al. 2015) is maximized at a higher P:C ratio. Assuming similar nutritional requirements exist in *T. commodus*, it appears unlikely that males are attempting to maximize any one of these specific traits but obviously does not rule out the possibility that males are prioritizing other fitness‐enhancing traits. Alternatively, the sexes in *T. commodus* may be regulating their intake of nutrients to balance the expression of multiple, competing traits (Bunning et al. 2015). For example, the sexual divergence we observe in the RIP and the midpoint between the P:C ratio maximizing LS and DRE in males (1_P_:5.25_C_) and females (1_P_:4.5_C_) are biased in the same direction. However, in both sexes the RIP is considerably more P biased than the midpoint, suggesting that if males and females are indeed balancing the nutritional demands of multiple traits, they are doing so for more than LS and DRE. It is also possible that the suboptimal pattern of nutrient regulation we observe in the sexes is due to physiological constraints. Dietary assimilation, digestion, absorption, and utilization are all known to constrain feeding behavior in animals (e.g., Henson and Hallam 1995) and variation in gut morphology often drives the efficiency of these processes (e.g., Penry and Jumars 1990). If the choice between complex diets limits any of these processes or is limited by gut morphology, it may prevent the sexes from optimally regulating nutrient intake. However, given that the RIP in both sexes is either close to or above the intake of nutrients needed to maximize LS, DRE, and LRE (Fig. 1), it is unlikely that gut morphology alone is limiting the intake of nutrients under dietary choice in *T. commodus*. Finally, the crickets used in our experiments had been maintained for 10 generations on a laboratory diet with a P:C ratio of 1_P_:1.23_C_ and it is possible that this may have inadvertently biased our RIPs, as well as our nutritional landscapes and estimates of **G** under dietary choice. Although this is a limitation facing all laboratory studies using cultured animals, it is difficult to see exactly how this may drive the pattern of suboptimal nutrient regulation we document, especially given the relatively central location of this laboratory diet in our overall geometric array of experimental diets (Fig. S1). In summary, the reasons for suboptimal nutrient regulation remain a fundamental question in nutritional ecology and one that clearly is going to require further empirical work to understand in *T. commodus*.

Associate Editor: B. Hollis

Handling Editor: M. Noor

## Supporting information


**Appendix S1**. Composition of Artificial Diets.
**Appendix S2**. Multivariate Response Surface Approach Used to Characterize the Nutritional Landscapes for Life Span and Reproductive Effort.
**Appendix S3**. Sequential Model‐Building Approach to Compare the Nutritional Landscapes for Life Span, Daily Reproductive Effort, and Lifetime Reproductive Effort.
**Appendix S4**. Calculating the Angle (θ) between Nutritional Vectors and 95% Credible Intervals.
**Appendix S5**. Calculate Linear Nutritional Effects of P and C, Additive Genetic Variance–Covariance (**G**) Matrix, $\Delta \bar{z}$, $\Delta {\bar{z}}_{B\;=\;0}$, the Angle (θ) between $\Delta \bar{z}$ and Linear Selection Gradient, the *R* Measure of Constraint and the Corresponding 95% Confidence Intervals for Each Measure.
**Table S1**. Protein (P) and carbohydrate (C) composition of the 24 artificial diets used in our no‐choice feeding experiment (Experiment 1).
**Figure S1**. The distribution of the 24 artificial diets used in our no‐choice feeding experiment (Experiment 1).
