# Supplementary material for: Little evidence for intralocus sexual conflict over the optimal intake of nutrients for life span and reproduction in the black field cricket Teleogryllus commodus
Source: Evolution. 2017 Jul 12;71(9):2159–77. doi: 10.1111/evo.13299 (PMC5599978; doi:10.1111/evo.13299)
Supplement: Supplementary file 1 — Appendix S1. Composition of Artificial Diets. Appendix S2. Multivariate Response Surface Approach Used to Characterize the Nutritional Landscapes for Life Span and Reproductive Effort. Appendix S3. Sequential Model‐Building Approach to Compare the Nutritional Landscapes for Life Span, Daily Reproductive Effort, and Lifetime Reproductive Effort. Appendix S4. Calculating the Angle (θ) between Nutritional Vectors and 95% Credible Intervals. Appendix S5. Calculate Linear Nutritional Effects of P and C, Additive Genetic Variance–Covariance (G) Matrix, Δz¯, Δz¯B=0, the Angle (θ) between Δz¯ and Linear Selection Gradient, the R Measure of Constraint and the Corresponding 95% Confidence Intervals for Each Measure. Table S1. Protein (P) and carbohydrate (C) composition of the 24 artificial diets used in our no‐choice feeding experiment (Experiment 1). Figure S1. The distribution of the 24 artificial diets used in our no‐choice feeding experiment (Experiment 1). [file EVO-71-2159-s001.docx]

**Online Supplement**

**Appendix S1. Composition of Artificial Diets**

**Table S1.** Protein (P) and carbohydrate (C) composition of the 24 artificial diets used in our no-choice feeding experiment (Experiment 1). The total nutrient concentration in each diet is given as the sum of the percentage P and percentage C, with the remaining percentage consisting of indigestible crystalline cellulose plus micronutrients. The four diets used in our quantitative genetic experiment (Experiment 2) and choice feeding experiment (Experiment 3) are highlighted in bold.

|  | **Percentage Composition** | | |  |
| --- | --- | --- | --- | --- |
| Diet number | P | C | P+C | P:C ratio |
| 1 | 10 | 2 | 12 | 5:1 |
| **2** | **30** | **6** | **36** | **5:1** |
| 3 | 50 | 10 | 60 | 5:1 |
| **4** | **70** | **14** | **84** | **5:1** |
| 5 | 9 | 3 | 12 | 3:1 |
| 6 | 27 | 9 | 36 | 3:1 |
| 7 | 45 | 15 | 60 | 3:1 |
| 8 | 63 | 21 | 84 | 3:1 |
| 9 | 6 | 6 | 12 | 1:1 |
| 10 | 18 | 18 | 36 | 1:1 |
| 11 | 30 | 30 | 60 | 1:1 |
| 12 | 42 | 42 | 84 | 1:1 |
| 13 | 3 | 9 | 12 | 1:3 |
| 14 | 9 | 27 | 36 | 1:3 |
| 15 | 15 | 45 | 60 | 1:3 |
| 16 | 21 | 63 | 84 | 1:3 |
| 17 | 2 | 10 | 12 | 1:5 |
| 18 | 6 | 30 | 36 | 1:5 |
| 19 | 10 | 50 | 60 | 1:5 |
| 20 | 14 | 70 | 84 | 1:5 |
| 21 | 1.33 | 10.66 | 12 | 1:8 |
| **22** | **4** | **32** | **36** | **1:8** |
| 23 | 6.66 | 53.33 | 60 | 1:8 |
| **24** | **9.33** | **74.66** | **84** | **1:8** |

**Figure S1.** The distribution of the 24 artificial diets used in our no-choice feeding experiment (Experiment 1). The diets are distributed along 6 nutritional rails (solid, black lines), with 4 diets per rail that differ in total nutritional content. On each nutritional rail, the diets connected by the isocaloric lines (dashed, black lines) have equal total nutrition. The 4 diets marked with red symbols represent those used in diet pairs in our genetic and choice feeding experiments (Experiments 2 and 3). The blue symbol represents the diet (50% mixture of cat and rat food) provided to our laboratory cultures of *T. commodus*.

**Appendix S2. Multivariate response surface approach used to characterize the nutritional landscapes for lifespan and reproductive effort**

We used a multivariate response surface approach to examine the effects of P and C intake on our response variables (lifespan, daily reproductive effort and lifetime reproductive effort). This approach is based on the methodologies of Lande & Arnold ([Lande and Arnold, 1983](#_ENREF_4)). Prior to analysis, we standardized each response variable and nutrient intake to a mean of zero and standard deviation of one using a *Z*-transformation to ensure that our regression gradients were presented in the same scale. First, the following linear multiple regression model is fitted to estimate the linear gradients for P and C intake on the response variable:

$R=\alpha+\beta P+\beta C+\varepsilon$ (Eq.1)

where *R* is the response variable, a is the regression intercept, βs represent the partial regression gradients and ɛ is the random error component.

To estimate the nonlinear (i.e. quadratic and correlational) gradients for nutrient intake on the response variables, the following nonlinear multiple regression model was fitted:

$R=\alpha+\beta P+\beta C+\frac{\gamma}{2}P^{2}+\frac{\gamma}{2}C^{2}+\gamma PC+\varepsilon$ (Eq.2)

where $\frac{\gamma}{2}P^{2}$and $\frac{\gamma}{2}C^{2}$represent the quadratic gradients for P and C, respectively and $\gamma PC$represents the correlational gradient for these two macronutrients. For the quadratic gradients, a negative term indicates a peak on the nutritional landscape, whereas a positive term indicates a trough. The linear terms are included but not interpreted from Eq. 2: they are simply included so that the nonlinear effects can be examined when the linear effects have been removed.

**Appendix S3. Sequential model building approach to compare the nutritional landscapes for lifespan, daily reproductive effort and lifetime reproductive effort**

We used a sequential model building approach to assess whether the linear and nonlinear effects of protein and carbohydrate ingestion differed for our response variables ([Draper and John, 1988](#_ENREF_3), [Chenoweth and Blows, 2005](#_ENREF_2)). As our different response variables (lifespan, daily reproductive effort and lifetime reproductive effort) were measured in different scales, it was necessary to standardize them for statistical comparison. Prior to comparison, we therefore standardized each response variable and nutrient intake to a mean of zero and a standard deviation of one using a *Z*-transformation. We then included a dummy variable, response type (*RT*), in a reduced model containing only the standardized linear terms:

(Eq.1)

where *R* is our standardized response variables, *N_i_* refers to the intake of the *i*th nutrient, *n* represents the number of nutrients contained in the model and *ε* is the unexplained error. From (1), the unexplained (i.e. residual) sums of squares for this reduced model (*SS_r_*) was compared to the same quantity (*SS_c_*) from a second (complete) model that included all of the terms in (1) with the addition of the terms *α_i_N_i_RT* which represents the linear interaction of *RT* and the *i*th nutrient.

$R=\beta_{0}+\alpha_{0}RT+\sum_{i=1}^{n} \beta_{i}N_{i}+\sum_{i=1}^{n} \alpha_{i}N_{i}RT+\varepsilon$ (Eq.2)

A partial *F*-test ([Bowerman and O'Connell, 1990](#_ENREF_1)) was used to compare *SS_r_* and *SS_c_* from (Eq.1) and (Eq.2) respectively:

$F_{a,b}=\frac{(SS_{r}-SS_{c})/a}{SS_{c}/b}$ (Eq.3)

where *a* is the number of terms that differ between the reduced and complete model and *b* is the error degrees of freedom for *SS_c_*.

To test whether the quadratic effect of nutrient intake differed between response variables, the *SS_r_* from the reduced model:

$R=\beta_{0}+\alpha_{0}RT+\sum_{i=1}^{n} \beta_{i}N_{i}+\sum_{i=1}^{n} \alpha_{i}N_{i}RT+\sum_{i=1}^{n} \beta_{i}N_{i}^{2}+\varepsilon$ (Eq.4)

was compared to the *SS_c_* of the complete model:

$R=\beta_{0}+\alpha_{0}RT+\sum_{i=1}^{n} \beta_{i}N_{i}+\sum_{i=1}^{n} \alpha_{i}N_{i}RT+\sum_{i=1}^{n} \beta_{i}N_{i}^{2}+\sum_{i=1}^{n} \alpha_{i}N_{i}^{2}RT+\varepsilon$ (Eq.5)

using (Eq.3).

To test whether correlational effects of nutrient intake on response variables differed, the *SS_r_* from the reduced model:

(Eq.6)

was compared to the *SS_c_* of the complete model:

(Eq.7)

using (Eq.3).

In summary, the comparison of model (Eq.1) versus (Eq.2), (Eq.4) versus (Eq.5), and (Eq.6) versus (Eq.7) provides a test for the overall significance of the interaction between response type and the linear, quadratic and correlational effects of nutrient intake, respectively. Therefore, significant differences in these model comparisons (as detected with a partial *F*-test) demonstrate that the linear, quadratic and/or correlational effects of nutrient intake on the response variables differ, respectively. We also inspected the interaction of individual nutrients with the response variable terms from the full model (Eq.7) to determine which of the nutrients were responsible for the significance of the overall partial *F*-test.

**Appendix S4. Calculating the angle (*θ*) between nutritional vectors and 95% credible intervals**

We calculated the angle (***θ***) between the linear vectors for the two response variables being compared as:

$\theta= \cos^{-1} \left( \frac{a \cdot b}{\|a\|\|b\|} \right)$ (1)

where *a* is the linear effects of P and C intake in the first response variable being compared, *b* is the linear effects of these nutrients for the second response variable, $\left\| a \right\|= \sqrt{a \cdot a}$ and $\left\| b \right\|= \sqrt{b \cdot b}$. When ***θ*** = 0° the vectors are perfectly aligned and the optima for the two response variables reside in the same location in nutrient space, whereas ***θ*** = 180° represents the maximum possible divergence between these vectors. To determine the significance of ***θ***, we estimated the 95% credible interval (CI) of this angle using a Bayesian approach implemented in the ‘MCMCglmm’ package of R. Using the linear models from the response surface approach above (Appendix S2), Bayesian inference was used to generate posterior distributions for each response variable and these were used to estimate the 95% CI for ***θ***.

**Text S4.** Annotated R code used to estimate the angle (*θ*), and 95% CIs, between linear vectors for the effects of nutrients on lifespan, daily reproductive effort and lifetime reproductive effort

#Load the package (MCMCglmm)

library(MCMCglmm)

# read in nutritional data for the first trait (e.g. lifespan)

angle.data<-read.table("LS.txt",h=T)

attach(angle.data)

str(angle.data)

#define a non-informative prior

prior<-list(R=list(V=1,nu=0.02))

# str(angle.data) should give a column for LS, P intake and C intake)

# Bayesian linear regression to estimate beta for each variable

# posterior distribution based on 15200 estimates of each parameter:

angle.model.1<-MCMCglmm(LS~P+C-1,data=angle.data, prior=prior,nitt=400000,burnin=20000,thin=25)

# and again for second trait (e.g. daily reproductive effort):

angle.data2<-read.table("DRE.txt",h=T)

attach(angle.data2)

str(angle.data2)

angle.model.2<-MCMCglmm(DRE~P+C-1,data=data,prior=prior,nitt=400000,burnin=20000,thin=25)

#summary of models (check against response surface analysis)

summary(selection.model.1)

summary(selection.model.2)

angles<-numeric(15200)

# creates an empty vector the same length as the posterior distribution, in # which angle estimates for each row of the posterior

# distribution will be stored as follows:

for(i in 1:15200){

b.LS<- angle.model.1$Sol[i,1:2]

b.DRE<- angle.model.2$Sol[i,1:2]

# creates a vector of beta estimates for each variable for each row of the # posterior distribution (and the loop runs through all rows)

angles[i]<- acos((t(b.LS) %*% b.DRE) / ((sqrt(t(b.LS) %*% b.LS)) * (sqrt(t(b.DRE) %*% b.DRE)))) * (180/pi) }

# calculates the angles between lifespan and daily reproductive effort betas for each row of the posterior distribution

summary(angles)

# to examine angle estimates which are now stored in the vector called #'angles'

HPDinterval(as.mcmc(angles),0.95)

# provides the 1st and 3^rd^ quantiles which are functionally equivalent to

# to the 95% CIs. Used the median and 95% CIs in this thesis for theta

**Appendix S5. Calculate linear nutritional effects of P and C, additive genetic variance-covariance (G) matrix, $\boldsymbol{\Delta}\bar{\boldsymbol{z}}$, $\boldsymbol{\Delta}{\overline{\boldsymbol{z}}}_{\mathbf{B}\boldsymbol{=0}}$, the angle (*θ*) between $\boldsymbol{\Delta}\bar{\boldsymbol{z}}$ and linear selection gradient, the $\boldsymbol{R}$ measure of constraint and the corresponding 95% confidence intervals for each measure**

**Text S5.** Annotated R code. Note that only the code to calculate all measures for lifespan in males is used in this example. All measures including male lifetime reproductive effort and daily reproductive effort as well as all the female estimates have been calculated and shown in the final manuscript by adjusting the below R code as appropriate.

#Read in the data

Data <- as.data.frame(read.table(file="./Intralocus_Daily.txt", header=T))

#Change the first column from ID to Animal

names(Data)[1] <- "animal"

#Specify factors and numerics from the data, include NA's

Data$animal<-as.factor(Data$animal)

Data$Sex<-as.factor(Data$Sex)

Data$DietPair<-as.factor(Data$DietPair)

Data$Total_P_Eaten<-as.numeric(Data$Total_P_Eaten)

Data$Total_P_Eaten_Daily<-as.numeric(Data$Total_P_Daily)

Data$Total_C_Eaten<-as.numeric(Data$Total_C_Eaten)

Data$Total_C_Eaten_Daily<-as.numeric(Data$Total_C_Daily)

Data$Total_P_Eaten_Male<-as.numeric(Data$Total_P_Eaten_Male, na.rm = F)

Data$Total_P_Eaten_Male_Daily<-as.numeric(Data$Total_P_Male_Daily, na.rm=F)

Data$Total_P_Eaten_Female<-as.numeric(Data$Total_P_Eaten_Female, na.rm = F)

Data$Total_P_Eaten_Female_Daily<-as.numeric(Data$Total_P_Female_Daily, na.rm=F)

Data$Total_C_Eaten_Male<-as.numeric(Data$Total_C_Eaten_Male, na.rm = F)

Data$Total_C_Eaten_Male_Daily<-as.numeric(Data$Total_C_Male_Daily, na.rm=F)

Data$Total_C_Eaten_Female<-as.numeric(Data$Total_C_Eaten_Female, na.rm = F)

Data$Total_C_Eaten_Female_Daily<-as.numeric(Data$Total_C_Female_Daily, na.rm=F)

head(Data)

#Read in the raw data values to get trait means

RawData <- read.csv("RawData.csv", header=T)

names(RawData)

MaleP <- mean(RawData$Male_P, na.rm=T)

MaleC <- mean(RawData$Male_C, na.rm=T)

FemaleP <- mean(RawData$Female_P, na.rm=T)

FemaleC <- mean(RawData$Female_C, na.rm=T)

#Read in the pedigree file

#Specify columns as factors

Ped <- as.data.frame(read.table(file = "./qg_pedigree.txt", header = T))

for (x in 1:3) Ped[,x] <- as.factor(Ped[,x])

head(Ped)

#Load packages

library(MCMCglmm)

library(Matrix)

#Specify the prior for the MCMC model, G is the G Matrix and R is the Residual Matrix

prior1.1 <- list(G = list(G1 = list(V = diag(4), n = 1.002)), R = list(V = diag(4), n = 1.002))

#Specify the model structure

#Specify the structure of the data e.g. normal, poisson, gaussian

#Include the terms to compare, random effects, and covariance effects

#Specify number of iterations, thinning number and burnin number

#Specify the prior, pedigree and data

#Model 1.1 is the standard model with all variance and co-variances calculated

model1.1 <- MCMCglmm(cbind(Total_P_Eaten_Male_Daily, Total_C_Eaten_Male_Daily, Total_P_Eaten_Female_Daily, Total_C_Eaten_Female_Daily) ~ trait -1, random = ~us(trait):animal,

rcov = ~us(trait):units, family = rep("gaussian", times=4),

pedigree = Ped, data = Data, nitt=15000, thin = 50, burnin=100,

prior = prior1.1, verbose = T)

# This is a model test, it tests the validity of the variance/covariance

# matrix's posterior distributions

# Best result is to be close to 0

autocorr(model1.1$VCV)

#Save the G matrix to a .csv file

model1.1_G <- model1.1$VCV

write.csv(model1.1_G, file="model1.1_G.csv")

#Create an object for the variance covariance matrix of model_1.1

model_VCV<-model1.1$VCV [1:2998, 1:16]

#Set up Model 1.2 Where the off-diagonal elements of G are set to 0

data2 <- data.frame(Data$animal, Data$Sex, Data$Total_P_Eaten_Daily, Data$Total_C_Eaten_Daily)

names(data2) <- c("animal", "sex", "Total_P", "Total_C")

data2$animal <- as.factor(data2$animal)

data2$sex <- as.factor(data2$sex)

prior1.4<-list(R = list(V = diag(2), n = 1.002), G=list(G1=list(V=diag(2), nu=1.002), G2=list(V=diag(2), nu=1.002)))

model1.2 <- MCMCglmm(cbind(Total_P, Total_C) ~ trait -1, random = ~us(trait:at.level(sex,"M")):animal + us(trait:at.level(sex, "F")):animal, rcov = ~us(trait):units, family = rep("gaussian", times=2),

pedigree=Ped, data = data2, nitt=15000, thin = 50, burnin=100,

prior=prior1.4, verbose = T)

#Create an object for the variance covariance matrix of model_1.2

model_1.2_VCV<-model1.2$VCV [1:2998, 1:8]

# Read in the data for Male Beta

# Bayesian linear regression to estimate beta for each male trait, produces

# posterior distribution for each parameter:

Male.Data <- read.table("Male_Beta.txt", header=T)

str(Male.Data)

# Beta for Male Lifespan

modelBM1 <- MCMCglmm(Z_MLS ~ Z_MP + Z_MC-1, data = Male.Data, nitt=15000, thin=50, burnin=100)

summary(modelBM1)

# Read in data for Female Beta

# Bayesian linear regression to estimate beta for each female trait,

# produces posterior distribution for each parameter:

Female.Data <- read.table("Female_Beta.txt", header=T)

str(Female.Data)

# Beta for Female Lifespan

modelBF1 <- MCMCglmm(Z_FLS ~ Z_FP + Z_FC-1, data = Female.Data, nitt=15000, thin=50, burnin=100)

summary(modelBF1)

#Create a vector the same life as the posterior and fill it with 0's to represent no intersexual correlation

x = 298

MP_FP_Blank <- rep(0, x)

MP_FC_Blank <- rep(0, x)

MC_FP_Blank <- rep(0, x)

MC_FC_Blank <- rep(0, x)

FP_MC_Blank <- rep(0, x)

FC_MC_Blank <- rep(0, x)

#Calculation for delta z bar

#Calculate deltaZ for Male Protein_LS

MP_LS_delta_z<-numeric(298)

# creates an empty vector the same length as the posterior distribution #((nitt-burnin)/thin) to calculate length of vector

# distribution will be stored as follows:

# Fill the empty vector

# Filled from the variance/covariance matrix and beta

# This fills the empty vector in row order but MCMC created the rows

# randomly during its iterative run

#Confirm this with a simple linear regression on one column of the

# posterior distribution outputs

for(i in 1:298){

MP_MP <- model1.1$VCV[i, 1]

MP_MC <- model1.1$VCV[i, 2]

MP_FP <- model1.1$VCV[i, 3]

MP_FC <- model1.1$VCV[i, 4]

MP_LS <- modelBM1$Sol[i, 1]

MC_LS <- modelBM1$Sol[i, 2]

FP_LS <- modelBF1$Sol[i, 1]

FC_LS <- modelBF1$Sol[i, 2]

#Calculate deltaz with below formula

MP_LS_delta_z[i]<- 0.5*((MP_MP*MP_LS)+(MP_MC*MC_LS)+(MP_FP*FP_LS)+(MP_FC*FC_LS))}

#Summary of deltaz including confidence

summary(MP_LS_delta_z)

HPDinterval(as.mcmc(MP_LS_delta_z),0.95)

#HPDinterval is an interval in which most of the distribution lies

#Calculate deltaZ for Male Carb_LS

MC_LS_delta_z<-numeric(298)

# creates an empty vector the same length as the posterior distribution

# distribution will be stored as follows:

for(i in 1:298){

MP_MC <- model1.1$VCV[i, 2]

MC_MC <- model1.1$VCV[i, 6]

FP_MC <- model1.1$VCV[i, 7]

FC_MC <- model1.1$VCV[i, 8]

MP_LS <- modelBM1$Sol[i, 1]

MC_LS <- modelBM1$Sol[i, 2]

FP_LS <- modelBF1$Sol[i, 1]

FC_LS <- modelBF1$Sol[i, 2]

MC_LS_delta_z[i]<- 0.5*((MP_MC*MP_LS)+(MC_MC*MC_LS)+(FP_MC*FP_LS)+(FC_MC*FC_LS)) }

summary(MC_LS_delta_z)

HPDinterval(as.mcmc(MC_LS_delta_z),0.95)

###########################################################################

#Calculate deltaZ_RB=0 for Male Protein_LS

MP_LS_delta_z_RB0<-numeric(298)

# creates an empty vector the same length as the posterior distribution

# distribution will be stored as follows:

for(i in 1:298){

MP_MP_RB0 <- model1.2$VCV[i, 1]

MP_MC_RB0 <- model1.2$VCV[i, 2]

MP_FP_RB0 <- MP_FP_Blank [i]

MP_FC_RB0 <- MP_FC_Blank [i]

MP_LS_RB0 <- modelBM1$Sol[i, 1]

MC_LS_RB0 <- modelBM1$Sol[i, 2]

FP_LS_RB0 <- modelBF1$Sol[i, 1]

FC_LS_RB0 <- modelBF1$Sol[i, 2]

MP_LS_delta_z_RB0[i]<- 0.5*((MP_MP_RB0 * MP_LS_RB0)+(MP_MC_RB0 * MC_LS_RB0)+(MP_FP_RB0 * FP_LS_RB0)+(MP_FC_RB0 * FC_LS_RB0))}

summary(MP_LS_delta_z_RB0)

HPDinterval(as.mcmc(MP_LS_delta_z_RB0),0.95)

#Calculate delta_Z_RB=0 for Male Carb_LS

MC_LS_delta_z_RB0 <-numeric(298)

# creates an empty vector the same length as the posterior distribution

# distribution will be stored as follows:

for(i in 1:298){

MP_MC_RB0 <- model1.2$VCV[i, 2]

MC_MC_RB0 <- model1.2$VCV[i, 4]

FP_MC_RB0 <- FP_MC_Blank [i]

FC_MC_RB0 <- FC_MC_Blank [i]

MP_LS_RB0 <- modelBM1$Sol[i, 1]

MC_LS_RB0 <- modelBM1$Sol[i, 2]

FP_LS_RB0 <- modelBF1$Sol[i, 1]

FC_LS_RB0 <- modelBF1$Sol[i, 2]

MC_LS_delta_z_RB0[i]<- 0.5*((MP_MC_RB0 * MP_LS_RB0)+(MC_MC_RB0 * MC_LS_RB0)+(FP_MC_RB0 * FP_LS_RB0)+(FC_MC_RB0 * FC_LS_RB0))}

summary(MC_LS_delta_z_RB0)

HPDinterval(as.mcmc(MC_LS_delta_z_RB0),0.95)

######## Calculate the Angle between Delta Z and Beta for Male LS

#Create a vector of n-length for the sum of Betas squared

Sum_Beta_LS<-numeric(298)

for(i in 1:298){

MP_LS <- modelBM1$Sol[i, 1]

MC_LS <- modelBM1$Sol[i, 2]

Sum_Beta_LS[i]<- ((MP_LS * MP_LS) + (MC_LS * MC_LS))}

#Calculate the square root of the Beta squared

Sqrt_Sum_Beta_LS <- sqrt(Sum_Beta_LS)

#Calculate delta_Z squared

MP_LS_squared <- (MP_LS_delta_z * MP_LS_delta_z)

MC_LS_squared <- (MC_LS_delta_z * MC_LS_delta_z)

#Sum of the delta_Z squared

Sum_Delta_z_LS_squared <- MP_LS_squared + MC_LS_squared

#Square root of sum of delta_z squared

Sqrt_Sum_Delta_z_LS <- sqrt(Sum_Delta_z_LS_squared)

#Calculate delta_Z * beta

Z_B1 <- MP_LS_delta_z * MP_LS

Z_B2 <- MC_LS_delta_z * MC_LS

#Sum of delta_Z * beta

Sum_Z_B_LS <- (Z_B1 + Z_B2)

#Calculate R - Sum of delta_z * beta divided by square root of sum delta_z # * square root of sum beta

r_LS <- Sum_Z_B_LS/(Sqrt_Sum_Delta_z_LS*Sqrt_Sum_Beta_LS)

#Calcuate the angle of r

angle_LS <- acos(r_LS) * (180/pi)

#Summary of angle data

summary(angle_LS)

HPDinterval(as.mcmc(angle_LS),0.95)

############Calculate the R value using delta_Z and delta_Z when intersex

# correlation is 0 for Lifespan

# R=0 the adaptation is comletely stalled by genetic correlation

# R=0.5 then the covariance structure causes the fitness of the mean

# phenotype to increase only 50% as quickly as expected if traits

# were genetically independent

# R=2 then genetic covariances accelerate evolution such that adaptation

# occurs twice as fast as expected under genetic independence

#Male Protein and Lifespan

MPLS_R <- function(MPLS_R){

MPdZLS <- sample(MP_LS_delta_z, size=1, replace=T)

MPdZ0LS <- sample(MP_LS_delta_z_RB0, size=1, replace=T)

MPLSdWC <- ((MaleP + MPdZLS) - MaleP)

MPLSdWI <- ((MaleP + MPdZ0LS) - MaleP)

MPLSR <- (MPLSdWC/MPLSdWI)

return(MPLSR)}

MPLS__R <- replicate(1000, MPLS_R())

summary(MPLS__R)

HPDinterval(as.mcmc(MPLS__R),0.95)

#Male Carb and Lifespan

MCLS_R <- function(MCLS_R){

MCdZLS <- sample(MC_LS_delta_z, size=1, replace=T)

MCdZ0LS <- sample(MC_LS_delta_z_RB0, size=1, replace=T)

MCLSdWC <- ((MaleC + MCdZLS) - MaleC)

MCLSdWI <- ((MaleC + MCdZ0LS) - MaleC)

MCLSR <- (MCLSdWC/MCLSdWI)

return(MCLSR)}

MCLS__R <- replicate(1000, MCLS_R())

summary(MCLS__R)

HPDinterval(as.mcmc(MCLS__R),0.95)

**References**

Bowerman, B. L., and R. T. O’Connell. 1990. Linear statistical models:an applied approach*.* Duxbury Press, Belmont, CA, USA.

Chenoweth, S. F., and M. W. Blows. 2005. Contrasting mutual sexual selection on homologous signal traits in *Drosophila serrata*. Am. Nat. 165**,** 281-289.

Draper, N. R., and J. A. John. 1988. Response-surface designs for quantitative and qualitative variables. Technometircs 30**,** 423-428.

Lande, R., and S. J. Arnold. 1983. The Measurement of Selection on Correlated Characters. Evolution*,* 37**,** 1210-1226.
